# Nanophotonic Chirality Transfer to Dielectric Mie Resonators

**DOI:** 10.1021/acs.nanolett.3c00739

**Published:** 2023-05-01

**Authors:** Ershad Mohammadi, T. V. Raziman, Alberto G. Curto

**Affiliations:** † Department of Applied Physics and Eindhoven Hendrik Casimir Institute, Eindhoven University of Technology, 5600MB Eindhoven, The Netherlands; ‡ Photonics Research Group, Ghent University-imec, 9052 Ghent, Belgium; § Center for Nano- and Biophotonics, Ghent University, 9052 Ghent, Belgium

**Keywords:** chiral sensing, circular dichroism, optical chirality, high-index dielectric metasurfaces

## Abstract

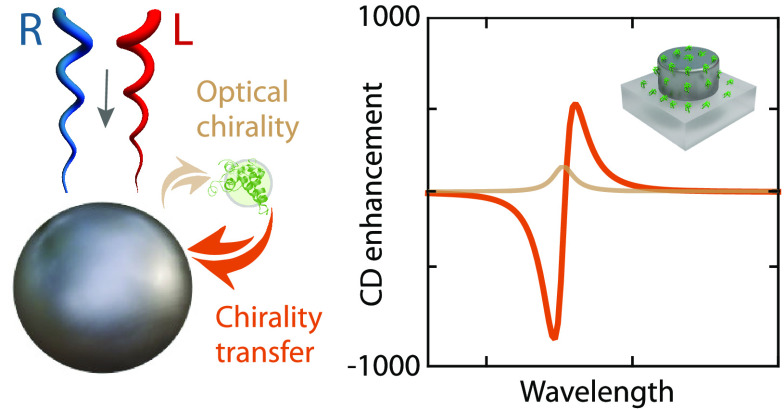

Nanophotonics can boost the weak circular dichroism of chiral molecules. One mechanism for enhanced chiral sensing relies on using a resonator to create fields with high optical chirality at the molecular position. Here, we elucidate how the reverse interaction between molecules and the resonator, called chirality transfer, can produce stronger circular dichroism. The chiral analyte modifies the electric and magnetic dipole moments of the resonator, imprinting a chiral response on an otherwise achiral resonance. We demonstrate that silicon nanoparticles and metasurfaces tailored for chirality transfer generate chiroptical signals orders of magnitude higher than the contribution from optical chirality alone. We derive closed-form equations for the dependence of chirality transfer on molecular chirality, molecule–resonator distance, and Mie coefficients. We propose a dielectric metasurface for a 900-fold circular dichroism enhancement on the basis of these principles. Finally, we identify a fundamental limit to chirality transfer. Our findings thus establish key concepts for nanophotonic chiral sensing.

Chiral molecules exist in right- and left-handed forms. The biological functionality of these enantiomers depends on their handedness, which makes chiral sensing crucial to medicine and biochemistry.^1^ Circular dichroism (CD) spectroscopy reports on molecular handedness through the differential absorption of circularly polarized light.^2^ However, it is typically very weak, particularly at low concentrations of chiral molecules. Nanophotonics provides a solution for overcoming this limitation and increasing sensitivity.

One physical mechanism for nanophotonic CD enhancement is based on increasing the optical chirality of light, defined as , where **E** and **H** are the complex electric and magnetic fields at the molecular position,^3,4^ and *k*_0_ and *c*_0_ are the wavenumber and speed of light in free space, respectively. Optical chirality can be enhanced beyond its value for circularly polarized plane waves. A possible route to such superchiral fields exploits the near field of metallic, dielectric, or hybrid nanoresonators.^5−13^ In this approach, CD enhancement is proportional to the optical chirality produced by the resonator at the position of each chiral molecule. Averaging over the chiral analyte volume is needed to obtain the total chiral response of the system.^8^ It is challenging to obtain large average enhancements because high optical chirality usually occurs in hot spots with limited volumes.

An alternative mechanism for enhancing chiral light–matter interaction is the transfer of the chiral response from the molecules to a nearby nanophotonic structure. We denominate this physical process as chirality transfer because CD is endowed upon an achiral resonance through the interaction of the chiral molecules with the nanoresonator modes. Such back-action of the chiral molecules on the resonator induces a chiroptical response at the wavelength of the nanophotonic resonance through dipole–dipole interactions. Another intuitive picture of chirality transfer is that the presence of the chiral molecules perturbs the local fields at the position of the achiral resonator, which would be otherwise equal under excitation with right- and left-handed circular polarizations. The potential of this mechanism has been demonstrated for metallic nanostructures exhibiting electric resonances.^14−19^ On the contrary, dielectric resonators now give access to both electric and magnetic resonances with tunable amplitudes, phases, and orientations. The versatility of their electric and magnetic dipolar moments has been exploited to maximize optical chirality^8−13^ and could instead be tailored for optimal chirality transfer.

Importantly, regardless of the material platform for nanophotonic chiral sensing, the CD of the combined system of molecules and nanostructures will most generally arise from the combined effects of the optical chirality and chirality transfer mechanisms. However, most studies thus far have focused on only one mechanism without considering both consistently, which has been a source of the mismatch between experimental results and theoretical predictions.^20−22^ Therefore, an incomplete understanding of the underlying physics responsible for chirality transfer still limits the rational design of nanophotonic platforms for chiral sensing.^23−26^

Here, we introduce chirality transfer to dielectric nanoresonators for CD enhancement orders of magnitude higher than what can be achieved by the optical chirality mechanism alone. By taking into account both effects consistently in dielectric systems with different geometries, we demonstrate that chirality transfer can dominate the total CD while the contribution of optical chirality is negligible. We derive analytical expressions for chirality transfer to dielectric nanospheres, elucidating its dependence on molecular chirality, distance, and dipolar Mie coefficients. We find that the coexistence of electric and magnetic resonances in the resonator can lead to a high degree of chirality transfer. Furthermore, we decompose chirality transfer into electric and magnetic contributions and identify the dominance of magnetic chirality transfer in high-refractive index nanostructures. We also propose a practical design for a dielectric metasurface with a 900-fold CD enhancement based on chirality transfer. Finally, we derive closed-form expressions for the fundamental upper limits of chirality transfer. Our findings thus specify the boundaries and differences between the chirality transfer and optical chirality mechanisms for nanophotonic CD enhancement.

## Chirality Transfer to Mie Resonators

**Figure 1:**
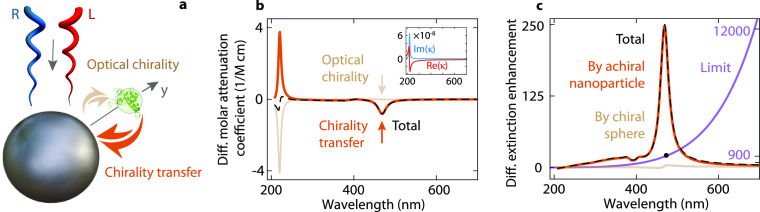
Chirality transfer from a chiral sphere to an achiral dielectric particle. (a) A small chiral sphere is located in the near field of an achiral nanoresonator. The system is illuminated by right- or left-handed circularly polarized plane waves. The achiral nanoparticle can enhance the circular dichroism of the chiral sphere due to an increase in local optical chirality (brown arrow). Conversely, the chiral sphere can perturb the response of the nanoparticle through chirality transfer (orange). (b) Differential molar attenuation coefficients and (c) differential extinction enhancements of the chiral sphere (brown), the achiral nanoparticle (orange), and the combined system (dashed black), including the upper limit of differential extinction enhancement (purple). The inset shows the real (red) and imaginary (blue) parts of the Pasteur parameter of the chiral sphere.

To investigate chirality transfer analytically, we first consider a small sphere made of a chiral material close to a high-index dielectric sphere (Figure 1a). Their radii are 5 and 50 nm and the center-to-center distance (*l*) is 60 nm. The achiral nanoresonator is made of silicon with realistic dispersion^27^ and is located at the origin of the coordinate system, whereas the chiral sphere is along the *y*-axis. The system is excited by a plane wave propagating along the *z*-axis with alternating right- and left-handed circular polarizations. The constitutive equations for a chiral medium include the Pasteur parameter (κ), which accounts for the coupling between the induced electric and magnetic dipoles through  and .^7^ We describe the permittivity and Pasteur parameter of the chiral sphere using two Lorentzian models with a molecular resonance at λ_0_ = 220 nm (Section S1 of the Supporting Information). To calibrate the Lorentzian parameters, we consider typical values for the differential (Δε) and the mean (ε̅) molar attenuation coefficients of the chiral analyte at molecular resonance.^2,15^ We then use the calibrated Pasteur parameter (Section S7) displayed in the inset of Figure 1b.

We analyze the optical response of the system in the electric–magnetic dipole approximation, where each particle is replaced by an electric and a magnetic dipole.^28,29^ The dipole moments induced in the particles arise from their polarizabilities and the local fields at their positions, consisting of the superposition of the incident field plus the scattered field due to the other particle. To derive the polarizabilities of the chiral sphere and the achiral nanoparticle, we exploit the quasi-static and exact Mie solutions, respectively.^30,31^ Then, the dipolar response of the coupled spheres is described by a self-consistent system of equations whose solution provides the induced dipole moments for right- and left-handed circularly polarized illuminations (Section S1). Applying free-space dyadic Green’s functions to such dipole moments, we find the total field at a given point in space. We are interested in the absorption of the nanoparticle for each excitation polarization in the presence of the chiral sphere, which is given by , where **E** and **H** are the total electric and magnetic fields over a spherical surface *S* enclosing the nanoparticle. We obtain the differential absorbed power by the nanoparticle as Δ*P*_abs_ = Δ*P*_ext_ – Δ*P*_sca_, where Δ*P*_ext_ and Δ*P*_sca_ are the differential extinct and scattered powers by the nanoparticle, expressed as (Section S2)
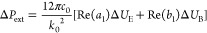
1
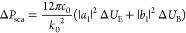
2where the terms  and  are the differential changes in time-averaged electric and magnetic energy densities at the center of the nanoparticle. *a*_1_ and *b*_1_ are the electric and magnetic dipolar coefficients of the Mie expansion of the achiral nanoparticle.^31^ In the absence of the chiral sphere, the chiroptical response of the achiral nanoparticle is zero due to symmetry (Δ*U*_E_ = Δ*U*_B_ = 0). The presence of the chiral sphere gives rise to non-zero differential extinct, scattered, and absorbed powers for the achiral nanoparticle. We define then the differential molar attenuation coefficient as Δε = 2.6157 × 10^20^Δσ, where Δσ = Δ*P*_ext_/*S*_in_ is the differential extinction cross section in square centimeters and *S*_in_ is the incident power density of the circularly polarized plane wave in watts per square centimeter (Section S1).

We obtain the differential attenuation coefficient of the chiral sphere (Figure 1b, brown line) and the dielectric nanoparticle (orange), which are due to optical chirality and chirality transfer, respectively. The total differential attenuation coefficient (black) has an induced peak at visible wavelengths. Its origin is chirality transfer, which contributes significantly more than optical chirality to the appearance of the peak. The observation of enhanced CD dominated by chirality transfer is in contrast to previous studies that focused on maximizing the average value of optical chirality near dielectric nanostructures.^9,10,21^ We also observe a cancellation effect around the molecular resonance, where chirality transfer reduces the intrinsic differential absorption of the chiral sphere. This effect becomes important if the goal is to increase CD at resonance in the ultraviolet.^32^

Assuming that the chiral sphere is very small and using the near-field symmetry conditions for right- and left-handed circularly polarized excitations, we obtain analytical expressions for the differential energy densities Δ*U*_E_ and Δ*U*_B_, and replace them in eqs 1 and 2. Then, by normalizing the differential extinct and scattered powers to the differential extinct power of the chiral sphere in free space, we obtain the differential extinction and scattering enhancements (Section S3):
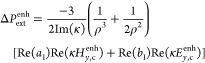
3
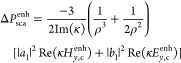
4where ρ = *k*_0_*l* is the distance parameter. The electric and magnetic field enhancements  and  indicate the enhancement in the *y*-components of the fields at the location of the chiral sphere. These local fields can be expressed in terms of the incident plus the dipolar fields of the nanoparticle. Assuming that the dipolar fields are dominant,^13^ we derive a fundamental limit *F* for  far from the molecular resonance (Section S5):
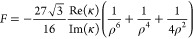
5where we assumed an upper limit of unity for the Mie coefficients.^33^ The differential extinction enhancement by the nanoparticle (Figure 1c, orange line) and its fundamental limit (purple) show that chirality transfer increases the differential extinction 250 times at λ = 468 nm, which reaches almost 30% of the predicted fundamental limit at that wavelength (*F* = 885). For comparison, we also calculate the differential extinction enhancement of the chiral sphere (brown) given by *C*_c_/*C*_inc_, where *C*_c_ and *C*_inc_ are the optical chirality values at the chiral sphere position and the incident field, respectively. The maximum enhancement due to optical chirality is 4.38, which is negligible compared to the chirality transfer contribution.

## Electric and Magnetic Contributions to Chirality Transfer

**Figure 2:**
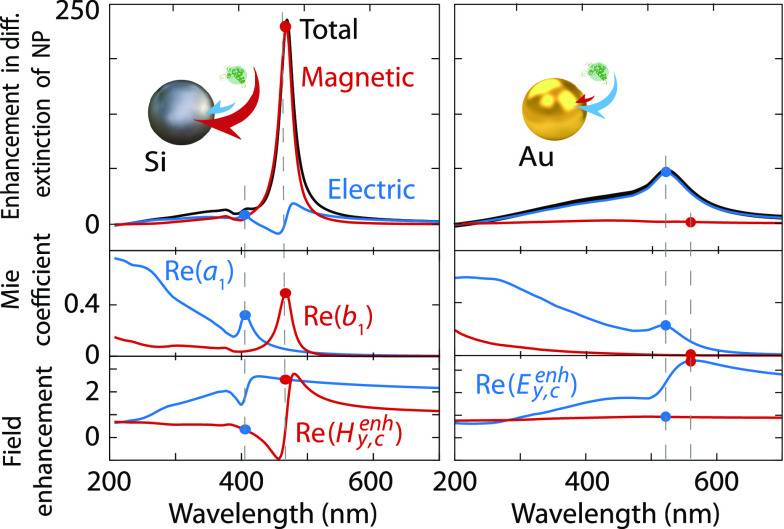
Electric and magnetic contributions to chirality transfer. Chirality transfer to the dielectric nanoparticle in Figure 1a (left) and a metallic nanoparticle with the same radius (right). Mie coefficients (real part) and field enhancements (real part of the *y*-components) at the location of the chiral sphere (bottom). Electric and magnetic quantities are shown as blue and red lines, respectively, where the dots indicate pairs of quantities contributing to electric and magnetic chirality transfer at a given wavelength.

To shed light on the origin of such enhancement due to chirality transfer, we decompose the differential extinction enhancement in eq 3 into electric and magnetic contributions. Far from the molecular resonance, where |Re(κ)| ≫ |Im(κ)|, we obtain the terms
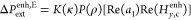
6
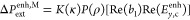
7where  and  are functions of the molecular chirality and distance, respectively. Physically, the electric (magnetic) contribution represents the part of chirality transfer due to the change in the electric (magnetic) energy density at the center of the nanoparticle. We display both contributions for a silicon sphere in Figure 2 (left) and compare them to those of a gold sphere of the same radius (right) .^34^ The magnetic contribution to chirality transfer is dominant for the dielectric nanoparticle, whereas a significantly smaller electric contribution prevails for the metallic sphere.

This result illustrates the need to tailor a nanophotonic resonator for optimal chirality transfer. The electric and magnetic fields and Mie coefficients must be carefully tuned. Dielectrics allow more favorable combinations, while metals impose strong limitations. We show the real parts of the Mie coefficient and the field components involved in eqs 6 and 7 in Figure 2 (bottom). For the dielectric particle, there is electric field enhancement at the peak of the magnetic Mie coefficient creating a high magnetic chirality transfer at λ = 468 nm. In contrast, in this system the combination of the electric Mie coefficient and magnetic field enhancement is not optimal for electric chirality transfer. For instance, the magnetic field enhancement is small at the electric resonance peak (λ = 406 nm). For the metallic sphere, the magnetic chirality transfer is small because there is no magnetic Mie resonance. The contribution of electric chirality transfer is not as high as the one of magnetic chirality transfer of the dielectric particle because there is almost no magnetic field enhancement at the peak of the electric resonance. Overall, the coexistence of electric and magnetic resonances plays a key role in maximizing chirality transfer.

## Chirality Transfer from a Chiral Shell to a Mie Resonator

**Figure 3:**
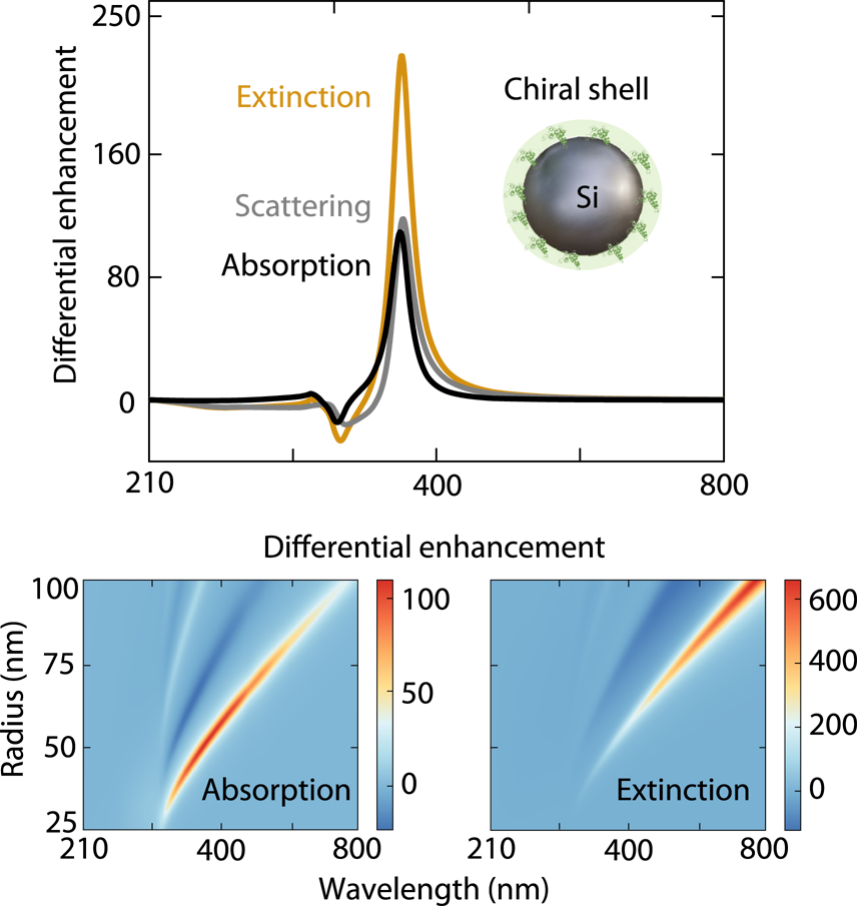
Chirality transfer from a chiral shell in extinction, scattering, and absorption. Enhancement in differential extinction (yellow), scattering (gray), and absorption (black) for a 50 nm silicon sphere compared to the differential absorption of a chiral shell in free space (top). Enhancements in differential absorption and extinction of the silicon sphere as a function of wavelength and nanoparticle radius (bottom). The shell thickness is 10 nm.

Next, we extend our theoretical framework to model chirality transfer for another realistic geometry, a dielectric sphere covered by a chiral shell of thickness δ_s_ (Figure 3). This geometry is spherically symmetric and thus allows us to prove that the existence of chirality transfer does not rely on a specific symmetry breaking of the overall configuration of the system. In the thin layer approximation (*k*_0_δ_s_ ≪ 1), we assume the local polarizing field of the shell to be the near field of the nanoparticle at the shell center. This problem can be regarded as an ensemble of small chiral spheres uniformly distributed around the dielectric sphere (Section S4). The collective effect of such spheres is obtained by integrating over the dipolar Green’s functions, yielding the differential extinction and scattering enhancements of the nanoparticle:
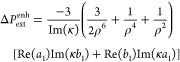
8
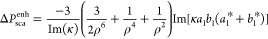
9where ρ = *k*_0_*r* is the distance parameter, *r* = *r*_i_ + δ_s_/2 is the mean distance of the chiral shell to the nanoparticle center, and *r*_i_ is the radius of the nanoparticle. The differential absorbed power inside the nanoparticle is Δ*P*_abs_ = Δ*P*_ext_ – Δ*P*_sca_. Note that, far from the molecular resonance, the averaged value of optical chirality over the chiral shell volume is related to Re(*a*_1_)Re(*b*_1_) + Im(*a*_1_)Im(*b*_1_), whereas chirality transfer behaves as Re(*a*_1_)Im(*b*_1_) + Im(*a*_1_)Re(*b*_1_). These relations imply that optical chirality is maximized when *a*_1_ = *b*_1_ = 1 for dual resonators,^13,35,36^ while chirality transfer is zero under this condition.

We compare the differential enhancements of extinction, scattering, and absorption in Figure 3 for a silicon sphere of radius *r*_i_ = 50 nm covered by a chiral shell of thickness δ_s_ = 10 nm. The enhancement values are normalized to the differential absorption of the chiral shell in free space. We find a 250-fold enhancement in differential extinction, similar to that for the chiral sphere in Figure 1c because the dominant part of chirality transfer for the chiral shell also comes from locations on the *x–y* plane where the field enhancements are maximum for illumination along the *z*-axis. Scattering and absorption contribute almost equally to the differential extinction enhancement.

To investigate the dependence of chirality transfer on nanoparticle size, we compute absorption and extinction as a function of wavelength and radius (Figure 3, bottom). Interestingly, there is an optimal radius around *r*_c_ = 50 nm for maximal absorption. Beyond this size, absorption decreases and scattering contributes more to extinction. This finding has important implications for chiral sensing because in experimentally relevant nanostructures, such as metasurfaces, the goal is to maximize differential absorption inside the resonators as we shall show next.

## Circular Dichroism Due to Chirality Transfer to a Metasurface

**Figure 4:**
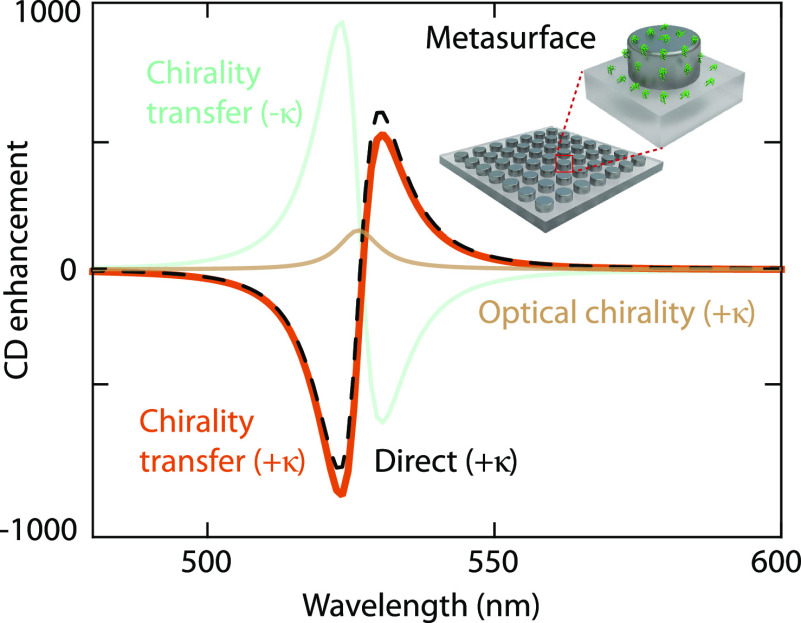
Chirality transfer can prevail over optical chirality in a dielectric metasurface. A conformal chiral layer covers an array of silicon disks. Contributions of chirality transfer (orange) and optical chirality (brown) to the total circular dichroism enhancement as directly simulated (black). Reversing the handedness of the chiral molecules changes the sign of the chirality transfer (green).

Chirality transfer also affects the circular dichroism measured in other practical chiral sensing schemes such as dielectric metasurfaces. To prove it, we rely on numerical simulations using COMSOL 5.5 (Section S8). We consider a silicon metasurface covered by a 10-nm-thick, conformal chiral layer (Figure 4, inset). The metasurface consists of disks with a radius of 80 nm and a height of 70 nm periodically arranged with a lattice constant of 320 nm on a glass substrate with a refractive index of 1.5. It is covered by a buffer solution with a refractive index of 1.33. To ensure the accuracy of the numerical simulations, we use a symmetric mesh^37^ and magnify the Pasteur parameter and the imaginary part of the permittivity of the chiral layer by a factor of 10^4^ (Figure 1b, inset) .^16^ We define CD as , where *T*_R_ and *T*_L_ denote the transmittance for right- and left-handed circularly polarized excitations, respectively.^2^ By neglecting scattering and assuming a small differential reflectance compared to differential transmittance (Section S9), we can express the CD enhancement as
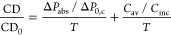
10where CD_0_ is the CD of the chiral film without nanostructures, *T* is the mean transmittance for both polarizations, Δ*P*_abs_ is the differential absorbed power inside the silicon disks, and Δ*P*_0,c_ is the same quantity inside the chiral film but in the absence of nanostructures. *C*_av_ and *C*_inc_ are the averaged value of optical chirality over the chiral film volume and the optical chirality of the incident field when the system is illuminated with either right- or left-handed circularly polarized light.

We can now decompose the total CD into the contributions from chirality transfer  and optical chirality  using eq 10. For this dielectric metasurface, we compare these two terms to the total CD directly calculated from the transmittances *T*_R_ and *T*_L_ (compare orange, brown, and black lines in Figure 4). The proposed system provides a 900-fold CD enhancement at λ = 525 nm dominated by chirality transfer with a considerably smaller contribution from optical chirality. When the molecular chirality is reversed through a sign flip of the Pasteur parameter (green), the results confirm the reversibility of the CD when the chirality transfer mechanism determines the chiral enhancement.

## Fundamental Limits to Chirality Transfer

**Figure 5:**
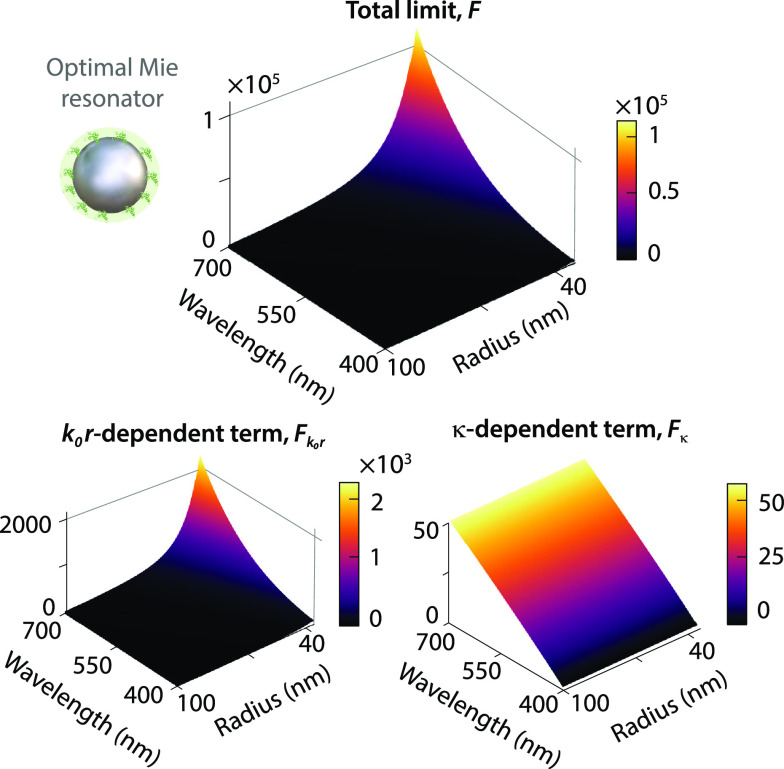
Fundamental limit of chirality transfer for an achiral nanoparticle surrounded by a thin chiral shell. Total limit of the differential extinction enhancement as a function of wavelength and nanoparticle radius (top). Distance- and chirality-dependent contributions to the total fundamental limit (bottom).

So far, we have used silicon as a realistic material for the nanophotonic resonators. However, it does not satisfy the requirements to maximize chirality transfer according to Mie theory. To gain insight into the ideal resonator material and size, we return to the chiral shell–achiral nanosphere geometry in Figure 3 to analytically find a fundamental limit of chirality transfer. By combining the optical theorem that sets Re(*a*_1_, *b*_1_) ≥ |*a*_1_, *b*_1_|^2^ and the unity upper limit for the Mie coefficients,^33,38^ we retrieve a fundamental limit of the differential extinction enhancement in eq 8 (Section S6). Far from the molecular resonance, the limit for an optimal Mie resonator is
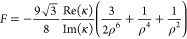
11which occurs when Re(*a*_1_) = |*a*_1_|^2^ = ^3^/_4_ and Re(*b*_1_) = |*b*_1_|^2^ = ^3^/_4_. This limit contains terms that depend on the particle radius through the molecule–resonator distance and on the wavelength dependence of the Pasteur parameter (Figure 5). The ideal Mie resonator for CD enhancement based on chirality transfer would thus operate at long wavelengths while having the smallest possible radius.

In conclusion, nanophotonic chirality transfer is a mechanism for circular dichroism enhancement that relies on modifying the dipolar response of an achiral resonator. We have demonstrated analytically and numerically that chirality transfer can create chiroptical signals that are orders of magnitude stronger than those arising from optical chirality for dielectric structures such as spheres and metasurfaces. Furthermore, we have established the combinations of electric and magnetic Mie coefficients and fields that give rise to chirality transfer through the Pasteur parameter. Consequently, we have identified a fundamental limit of chirality transfer to Mie resonances. Understanding this electrodynamic mechanism is essential because chirality transfer is complementary to optical chirality for circular dichroism enhancement. In general, both mechanisms can coexist and should be assessed on an equal footing. Our findings can be directly applied to explain recent experiments^21^ and are necessary for the rational and consistent design of nanophotonic platforms for ultrasensitive circular dichroism.

## Supporting Information Available

Analytical investigation of a chiral sphere–achiral nanoparticle system using the coupled electric–magnetic dipole approximation; extinction, scattering, and absorption for a chiral sphere–achiral nanoparticle system; transfer of chirality from a small chiral sphere to an achiral nanoparticle; transfer of chirality from a chiral shell to an achiral nanoparticle; fundamental limits of chirality transfer for a small chiral sphere near an achiral nanoparticle; fundamental limits of chirality transfer for an achiral nanoparticle covered by a thin chiral shell; Beer–Lambert law and calibration of the Lorentzian model; numerical simulations; and definition of circular dichroism (PDF)
