# Supplementary material for: Nanophotonic Chirality Transfer to Dielectric Mie Resonators
Source: Nano Lett. 2023 May 1;23(9):3978–84. doi: 10.1021/acs.nanolett.3c00739 (PMC10176573; doi:10.1021/acs.nanolett.3c00739)
Supplement: Supplementary file 1 — nl3c00739_si_001.pdf [file nl3c00739_si_001.pdf]

Supporting Information

Nanophotonic Chirality Transfer to Dielectric Mie Resonators

Ershad Mohammadi[1], T.V. Raziman[1], and Alberto G. Curto[1,2,3]

[1] Department of Applied Physics and Eindhoven Hendrik Casimir Institute,

Eindhoven University of Technology, Eindhoven, The Netherlands

[2] Photonics Research Group, Ghent University-imec, Ghent, Belgium

[3] Center for Nano- and Biophotonics, Ghent University, Ghent, Belgium

Contents:

Supporting Section S1. Analytical investigation of a chiral sphere-achiral nanoparticle system using the coupled electric-magnetic dipole approximation

Supporting Section S2. Extinction, scattering, and absorption for a chiral sphere-achiral nanoparticle system

Supporting Section S3. Transfer of chirality from a small chiral sphere to an achiral nanoparticle

Supporting Section S4. Transfer of chirality from a chiral shell to an achiral nanoparticle

Supporting Section S5. Fundamental limits of chirality transfer for a small chiral sphere near an achiral nanoparticle

Supporting Section S6. Fundamental limits of chirality transfer for an achiral nanoparticle covered by a thin chiral shell

Supporting Section S7. Beer-Lambert law and calibration of the Lorentzian model

Supporting Section S8. Numerical simulations

Supporting Section S9. Circular dichroism definition

Supporting Section S1. Analytical investigation of a chiral sphere-achiral nanoparticle system using the coupled electric-magnetic dipole approximation

The system under study in this section consists of a spherical nanoparticle of radius R_i located at the center of the coordinate system and placed at a distance l from a small chiral sphere of radius R_c (Figure S1). The chiral particle is located on the y-axis, and the whole system is excited with a circularly polarized plane wave propagating along the z-direction. The incident electric and magnetic fields are expressed as:

$$\boldsymbol{E}_{inc} = E_0 \exp(-ik_0 z)\, \hat{e}_R$$
$$\boldsymbol{H}_{inc} = \left(iE_0 / \eta_0\right)\exp(-ik_0 z)\, \hat{e}_R \qquad \text{(S1)}$$

where E_0 and η_0 are the electric field amplitude and the free-space wave impedance, respectively. k_0 is the free-space wavenumber and $\hat{e}_R = \left(\hat{x} - i\hat{y}\right)$ is the base vector for right-handed circular polarization.[1] The dipolar response of the system can be described by replacing each particle with an electric ($\boldsymbol{p}$; blue arrows in Figure S1 showing the three possible Cartesian components) and a magnetic dipole ($\boldsymbol{m}$; red arrows) pair.

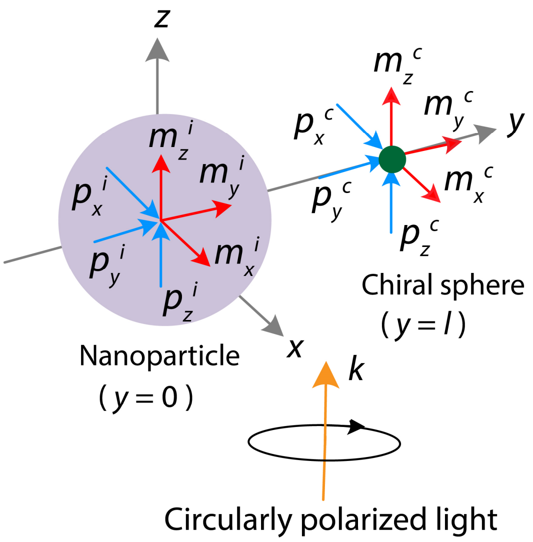

Figure S1. The system under study for investigating the transfer of chirality from a small chiral sphere ("*c*") to a spherical nanoparticle ("*i*"). The blue and red arrows indicate the components of the induced electric and the magnetic dipole moments in all directions, respectively.

The induced dipole moments in the nanoparticle and chiral sphere are related to their polarizabilities as:

$$\begin{pmatrix} \mathbf{p}^i \\ \mathbf{m}^i \end{pmatrix} = \begin{pmatrix} \alpha_e^i & \\ & \alpha_m^i \end{pmatrix} \begin{pmatrix} \mathbf{E}^i \\ \mathbf{H}^i \end{pmatrix} \qquad \text{(S2)}$$

and

$$\begin{pmatrix} \mathbf{p}^c \\ \mathbf{m}^c \end{pmatrix} = \begin{pmatrix} \alpha_{ee}^c & \alpha_{em}^c \\ \alpha_{me}^c & \alpha_{mm}^c \end{pmatrix} \begin{pmatrix} \mathbf{E}^c \\ \mathbf{H}^c \end{pmatrix} \qquad \text{(S3)}$$

respectively, where $\boldsymbol{E}$ and $\boldsymbol{H}$ are the local electric and magnetic fields originating from the incident field plus the dipolar fields caused by the other particle. The superscripts ("*i*") and ("*c*") refer to the position of the nanoparticle and the chiral sphere, respectively. The electric and the magnetic dipole polarizabilities of the nanoparticle are $\alpha_e^i = \left(-6\pi i \varepsilon_0 / k_0^3\right) a_1$ and $\alpha_m^i = \left(-6\pi i / k_0^3\right) b_1$, where a_1 and b_1 represent the electric and

the magnetic dipolar coefficients in the Mie expansion.[2] For the chiral sphere, we use the quasi-static polarizabilities as[3]:

$$\alpha_{ee}^{c} = 4\pi\varepsilon_0 R_c^3 \frac{\left(\mu_r^c + 2\right)\left(\varepsilon_r^c - 1\right) - \kappa^2}{\left(\mu_r^c + 2\right)\left(\varepsilon_r^c + 2\right) - \kappa^2} \tag{S4}$$

$$\alpha_{em}^{c} = 4\pi\varepsilon_0 \eta_0 R_c^3 \frac{-3i\kappa}{\left(\mu_r^c + 2\right)\left(\varepsilon_r^c + 2\right) - \kappa^2} \tag{S5}$$

$$\alpha_{me}^{c} = -\alpha_{em}^{c} / \mu_0 \tag{S6}$$

$$\alpha_{mm}^{c} = 4\pi R_c^3 \frac{\left(\mu_r^c - 1\right)\left(\varepsilon_r^c + 2\right) - \kappa^2}{\left(\mu_r^c + 2\right)\left(\varepsilon_r^c + 2\right) - \kappa^2} \tag{S7}$$

where ε_r^c and μ_r^c are the relative permittivity and the relative permeability of the chiral sphere. κ is the Pasteur parameter indicating molecular chirality. For non-magnetic materials, the relative permeability is always $\mu_r^c = 1$. The permittivity and the Pasteur parameter can be described by a Lorentzian model:[4]

$$\varepsilon_r^c = \varepsilon_b - \gamma \left(\frac{1}{\hbar\omega - \hbar\omega_0 + i\,\Gamma} - \frac{1}{\hbar\omega + \hbar\omega_0 + i\,\Gamma} \right) \tag{S8}$$

$$\kappa = \beta \left(\frac{1}{\hbar\omega - \hbar\omega_0 + i\,\Gamma} - \frac{1}{\hbar\omega + \hbar\omega_0 + i\,\Gamma} \right) \tag{S9}$$

where $\hbar$ is the reduced Planck's constant, $\omega_0 = 2\pi / \lambda_0$ is the angular frequency of the molecular resonance, Γ denotes the damping factor, which is considered to be 0.1 eV to give $FWHM \simeq 10$ nm. We assume a single optical transition for chiral molecules at wavelength $\lambda_0 = 220$ nm, and the background permittivity is considered as $\varepsilon_b = 1.33^2$. The coefficients γ and β relate to the strength of the absorption and the differential absorption, respectively. To calibrate these parameters, we consider typical values of $20\ \mathrm{M^{-1}\ cm^{-1}}$ and $10^4\ \mathrm{M^{-1}\ cm^{-1}}$ for the differential ($\Delta\varepsilon$) and the mean ($\bar{\varepsilon}$) molar attenuation coefficients of the chiral analyte at molecular resonance[5], which renders γ=1.8×10^{-5} eV and β=6.53×10^{-9} eV for a molar concentration of 1.6 mM (see Supporting Section S7).

By expanding the local fields in Equations S2 and S3 in terms of the incident and the dipolar fields, we can describe the electromagnetic interaction of dipole pairs in Figure S1 by the following self-consistent system of coupled equations:

$$\begin{aligned} \mathbf{p}^i &= \alpha_e^i \left(\boldsymbol{E}_{inc} + \overleftrightarrow{\boldsymbol{G}}_{EP} \cdot \mathbf{p}^c + \overleftrightarrow{\boldsymbol{G}}_{EM} \cdot \mathbf{m}^c \right) \\ \mathbf{m}^i &= \alpha_m^i \left(\boldsymbol{H}_{inc} + \overleftrightarrow{\boldsymbol{G}}_{HM} \cdot \mathbf{m}^c + \overleftrightarrow{\boldsymbol{G}}_{HP} \cdot \mathbf{p}^c \right) \end{aligned} \tag{S10}$$

$$\begin{aligned}\mathbf{p}^c &= \alpha_{ee}^c\left(\boldsymbol{E}_{inc} + \overleftrightarrow{\boldsymbol{G}}_{EP}\cdot\mathbf{p}^i + \overleftrightarrow{\boldsymbol{G}}_{EM}\cdot\mathbf{m}^i\right) + \alpha_{em}^c\left(\overleftrightarrow{\boldsymbol{G}}_{HP}\cdot\mathbf{p}^i + \overleftrightarrow{\boldsymbol{G}}_{HM}\cdot\mathbf{m}^i\right)\\ \mathbf{m}^c &= \alpha_{mm}^c\left(\boldsymbol{H}_{inc} + \overleftrightarrow{\boldsymbol{G}}_{HP}\cdot\mathbf{p}^i + \overleftrightarrow{\boldsymbol{G}}_{HM}\cdot\mathbf{m}^i\right) + \alpha_{me}^c\left(\overleftrightarrow{\boldsymbol{G}}_{EP}\cdot\mathbf{p}^i + \overleftrightarrow{\boldsymbol{G}}_{EM}\cdot\mathbf{m}^i\right)\end{aligned} \tag{S11}$$

where $\overleftrightarrow{\boldsymbol{G}}_{EP}$ and $\overleftrightarrow{\boldsymbol{G}}_{HP}$ ($\overleftrightarrow{\boldsymbol{G}}_{EM}$ and $\overleftrightarrow{\boldsymbol{G}}_{HM}$) are the electric and the magnetic dyadic Green's functions for radiation of an electric (a magnetic) dipole source in free space, which are expressed as:

$$\overleftrightarrow{\boldsymbol{G}}_{EP}(\boldsymbol{r},\boldsymbol{r}_0) = \frac{k_0^3}{\varepsilon_0}\frac{\exp(-ik_0R)}{4\pi k_0R}\left\{\left(1+\frac{1}{ik_0R}-\frac{1}{k_0^2R^2}\right)\overleftrightarrow{I} - \left(1+\frac{3}{ik_0R}-\frac{3}{k_0^2R^2}\right)\hat{R}\hat{R}\right\} \tag{S12}$$

$$\overleftrightarrow{\boldsymbol{G}}_{HP}(\boldsymbol{r},\boldsymbol{r}_0) = c_0k_0^3\frac{\exp(-ik_0R)}{4\pi k_0R}\left(1+\frac{1}{ik_0R}\right)\hat{R}\times\overleftrightarrow{I} \tag{S13}$$

$$\overleftrightarrow{\boldsymbol{G}}_{EM}(\boldsymbol{r},\boldsymbol{r}_0) = -\eta_0k_0^3\frac{\exp(-ik_0R)}{4\pi k_0R}\left(1+\frac{1}{ik_0R}\right)\hat{R}\times\overleftrightarrow{I} \tag{S14}$$

$$\overleftrightarrow{\boldsymbol{G}}_{HM}(\boldsymbol{r},\boldsymbol{r}_0) = k_0^3\frac{\exp(-ik_0R)}{4\pi k_0R}\left\{\left(1+\frac{1}{ik_0R}-\frac{1}{k_0^2R^2}\right)\overleftrightarrow{I} - \left(1+\frac{3}{ik_0R}-\frac{3}{k_0^2R^2}\right)\hat{R}\hat{R}\right\} \tag{S15}$$

where the $\boldsymbol{r}$ and $\boldsymbol{r}_0$ vectors refer to the observation point and the dipole location, respectively. $R = |\boldsymbol{r} - \boldsymbol{r}_0|$ denotes the distance between the dipole source and the observation point. $\hat{R} = (\boldsymbol{r} - \boldsymbol{r}_0)/R$ is the unit vector pointing from the dipole towards the observation point. $\overleftrightarrow{I}$ is the unit dyad.[6] By putting these Green's functions in Equations S10 and S11, we can reformulate the self-consistent system for right-handed circularly polarized excitation (Equation S1) in the following matrix form:

$$\begin{pmatrix} 1 & 0 & -\alpha_e^i\left(k_0^3/\varepsilon_0\right)\zeta\left(k_0l\right) & 0 \\ -\alpha_{ee}^c\left(k_0^3/\varepsilon_0\right)\zeta\left(k_0l\right) & -\alpha_{em}^c k_0^3\zeta\left(k_0l\right) & 1 & 0 \\ 0 & 1 & 0 & -\alpha_m^i k_0^3\zeta\left(k_0l\right) \\ -\alpha_{me}^c\left(k_0^3/\varepsilon_0\right)\zeta\left(k_0l\right) & -\alpha_{mm}^c k_0^3\zeta\left(k_0l\right) & 0 & 1 \end{pmatrix}\begin{pmatrix} p_y^i \\ m_y^i \\ p_y^c \\ m_y^c \end{pmatrix} = \begin{pmatrix} -i\alpha_e^iE_0 \\ -i\alpha_{ee}^cE_0 + \alpha_{em}^cE_0/\eta_0 \\ \alpha_m^iE_0/\eta_0 \\ \alpha_{mm}^cE_0/\eta_0 - i\alpha_{me}^cE_0 \end{pmatrix} \tag{S16}$$

$$\begin{pmatrix} 1 & 0 & 0 & 0 & -\alpha_e^i\left(k_0^3/\varepsilon_0\right)\rho\left(k_0l\right) & 0 & 0 & -\alpha_e^i\eta_0k_0^3\ \tau\left(k_0l\right) \\ 0 & 1 & 0 & 0 & 0 & -\alpha_e^i\left(k_0^3/\varepsilon_0\right)\rho\left(k_0l\right) & \alpha_e^i\eta_0k_0^3\ \tau\left(k_0l\right) & 0 \\ 0 & 0 & 1 & 0 & 0 & \alpha_m^ic_0k_0^3\ \tau\left(k_0l\right) & -\alpha_m^ik_0^3\rho\left(k_0l\right) & 0 \\ 0 & 0 & 0 & 1 & -\alpha_m^ic_0k_0^3\ \tau\left(k_0l\right) & 0 & 0 & -\alpha_m^ik_0^3\rho\left(k_0l\right) \\ -\alpha_{ee}^c\left(k_0^3/\varepsilon_0\right)\rho\left(k_0l\right) & -\alpha_{em}^cc_0k_0^3\ \tau\left(k_0l\right) & -\alpha_{em}^ck_0^3\ \rho\left(k_0l\right) & \alpha_{ee}^c\eta_0k_0^3\ \tau\left(k_0l\right) & 1 & 0 & 0 & 0 \\ \alpha_{em}^cc_0k_0^3\ \tau\left(k_0l\right) & -\alpha_{ee}^c\left(k_0^3/\varepsilon_0\right)\rho\left(k_0l\right) & -\alpha_{ee}^c\eta_0k_0^3\ \tau\left(k_0l\right) & -\alpha_{em}^ck_0^3\ \rho\left(k_0l\right) & 0 & 1 & 0 & 0 \\ -\alpha_{me}^c\left(k_0^3/\varepsilon_0\right)\rho\left(k_0l\right) & -\alpha_{mm}^cc_0k_0^3\ \tau\left(k_0l\right) & -\alpha_{mm}^ck_0^3\rho\left(k_0l\right) & \alpha_{me}^c\eta_0k_0^3\ \tau\left(k_0l\right) & 0 & 0 & 1 & 0 \\ \alpha_{mm}^cc_0k_0^3\ \tau\left(k_0l\right) & -\alpha_{me}^c\left(k_0^3/\varepsilon_0\right)\rho\left(k_0l\right) & -\alpha_{me}^c\eta_0k_0^3\ \tau\left(k_0l\right) & -\alpha_{mm}^ck_0^3\rho\left(k_0l\right) & 0 & 0 & 0 & 1 \end{pmatrix} \begin{pmatrix} p_x^i \\ p_z^i \\ m_x^i \\ m_z^i \\ p_x^c \\ p_z^c \\ m_x^c \\ m_z^c \end{pmatrix} = \begin{pmatrix} \alpha_e^iE_0 \\ 0 \\ i\alpha_m^iE_0/\eta_0 \\ 0 \\ \alpha_{ee}^cE_0 + i\alpha_{em}^cE_0/\eta_0 \\ 0 \\ i\alpha_{mm}^cE_0/\eta_0 + \alpha_{me}^cE_0 \\ 0 \end{pmatrix} \quad \text{(S17)}$$

where $\rho\left(x\right) = \exp(-ix)\left(x^{-1} - ix^{-2} - x^{-3}\right)/4\pi$ and $\zeta\left(x\right) = \exp(-ix)\left(ix^{-2} + x^{-3}\right)/2\pi$ are functions related to the *xx*- and *yy*-components of the $\overleftrightarrow{\boldsymbol{G}}_{EP}$ / $\overleftrightarrow{\boldsymbol{G}}_{HM}$, respectively, while $\tau\left(x\right) = x\,\zeta\left(x\right)/2i$ is related to the *xz/zx*-components of the $\overleftrightarrow{\boldsymbol{G}}_{EM}$ / $\overleftrightarrow{\boldsymbol{G}}_{HP}$. Solving these systems of equations gives the induced dipole moments in all directions. The matrix system in Equations S16 and S17 shows that the *y*-components of the dipole moments (p_y^i , m_y^i , p_y^c , m_y^c , m_y) are decoupled from the dipole moments induced along the *x*- and *z*-directions. As we shall later see, these are the components that determine the dominant response of the system.

Supporting Section S2. Extinction, scattering, and absorption for a chiral sphere-achiral nanoparticle system

Now that the induced dipole moments for the RCP and the LCP excitations have been obtained, we can calculate the absorption to see how the presence of the chiral sphere gives rise to a non-zero differential absorption for the achiral nanoparticle (NP). To figure this out, we consider a closed surface *S* around the NP as shown in Figure S2 and calculate the total absorbed power inside the NP as:

$$P_{abs} = -\frac{1}{2}\int_S \mathrm{Re}\left(\boldsymbol{E} \times \boldsymbol{H}^*\right)\cdot d\boldsymbol{s} \qquad \text{(S18)}$$

where $\boldsymbol{E}$ and $\boldsymbol{H}$ are the total electric and magnetic fields over the surface *S*, which are expressed as:

$$\boldsymbol{E} = \boldsymbol{E}_{inc} + \boldsymbol{E}_p + \boldsymbol{E}_m \qquad \text{(S19)}$$

$$\boldsymbol{H} = \boldsymbol{H}_{inc} + \boldsymbol{H}_p + \boldsymbol{H}_m \qquad \text{(S20)}$$

where $\boldsymbol{E}_{inc}$ and $\boldsymbol{H}_{inc}$ are the incident electric and magnetic fields over the surface *S*. $\boldsymbol{E}_p$ and $\boldsymbol{H}_p$ ($\boldsymbol{E}_m$ and $\boldsymbol{H}_m$) are the dipolar electric the magnetic fields due to the electric dipole (magnetic dipole) of the nanoparticle, which are related to the dyadic Green's functions as $\boldsymbol{E}_p = \overleftrightarrow{\boldsymbol{G}}_{EP} \cdot \boldsymbol{p}$, $\boldsymbol{E}_m = \overleftrightarrow{\boldsymbol{G}}_{EM} \cdot \boldsymbol{m}$, $\boldsymbol{H}_p = \overleftrightarrow{\boldsymbol{G}}_{HP} \cdot \boldsymbol{p}$, and $\boldsymbol{H}_m = \overleftrightarrow{\boldsymbol{G}}_{HM} \cdot \boldsymbol{m}$. Here, for the dyadic Green's functions in Equations S12-S15, the dipole position is $\boldsymbol{r}_0 = 0$, and the observation point is always on the surface *S* with $\boldsymbol{r} = r\hat{\boldsymbol{r}}$, where r is the radius of the nanoparticle. For simplicity, in this section we dropped the superscript "*i*" for the nanoparticle. Substituting the total fields of Equation S19 and S20 in Equation S18 gives:

$$\begin{aligned} P_{abs} &= -\frac{1}{2}\int_S \mathrm{Re}\left(\boldsymbol{E}_{inc} \times \boldsymbol{H}_p^* + \boldsymbol{E}_p \times \boldsymbol{H}_{inc}^* + \boldsymbol{E}_{inc} \times \boldsymbol{H}_m^* + \boldsymbol{E}_m \times \boldsymbol{H}_{inc}^*\right) \cdot d\boldsymbol{s} \\ &-\frac{1}{2}\int_S \mathrm{Re}\left(\boldsymbol{E}_p \times \boldsymbol{H}_p^* + \boldsymbol{E}_m \times \boldsymbol{H}_m^* + \boldsymbol{E}_p \times \boldsymbol{H}_m^* + \boldsymbol{E}_m \times \boldsymbol{H}_p^*\right) \cdot d\boldsymbol{s} \end{aligned} \quad \text{(S21)}$$

which shows the total absorbed power of the nanoparticle in terms of the incident-dipolar (the first four terms in Equation S21) and fully dipolar (the second four terms in Equation S21) absorption. To obtain P_{abs} , we need to calculate the eight cross-product terms in Equation S21 separately. For brevity, here we only show the derivation for $\boldsymbol{E}_{inc} \times \boldsymbol{H}_p^*$ and $\boldsymbol{E}_p \times \boldsymbol{H}_p^*$ while the same approach can be used for other terms.

Using Green's functions $\overleftrightarrow{\boldsymbol{G}}_{EP}$ and $\overleftrightarrow{\boldsymbol{G}}_{HP}$ in Equations S12 and S13, the $\boldsymbol{E}_p \times \boldsymbol{H}_p^*$ cross product can be written as:

$$\begin{aligned} &\boldsymbol{E}_p \times \boldsymbol{H}_p^* = \\ &\left(\frac{c_0}{\varepsilon_0}\right)\left(\frac{k_0^3}{4\pi k_0 r}\right)^2\left(1-\frac{1}{ik_0 r}\right)\left\{\left(1+\frac{1}{ik_0 r}-\frac{1}{k_0^2 r^2}\right)\boldsymbol{p} \times \left(\hat{r} \times \boldsymbol{p}^*\right) - \left(1+\frac{3}{ik_0 r}-\frac{3}{k_0^2 r^2}\right)\left(\hat{r}\hat{r} \cdot \boldsymbol{p}\right) \times \left(\hat{r} \times \boldsymbol{p}^*\right)\right\} \end{aligned} \quad \text{(S22)}$$

Then, by exploiting the vectorial identities $\boldsymbol{p} \times \left(\hat{r} \times \boldsymbol{p}^*\right) = |\boldsymbol{p}|^2 \hat{r} - p_r \boldsymbol{p}^*$ and $\left(\hat{r}\hat{r} \cdot \boldsymbol{p}\right) \times \left(\hat{r} \times \boldsymbol{p}^*\right) = |p_r|^2 \hat{r} - p_r \boldsymbol{p}^*$, we can rewrite Equation S22 as follows:

$$\int_S \left(\boldsymbol{E}_p \times \boldsymbol{H}_p^*\right). d\boldsymbol{s} = \left(\frac{c_0}{\varepsilon_0}\right)\left(\frac{k_0^3}{4\pi k_0 r}\right)^2\left(1-\frac{1}{ik_0 r}\right)\left(1+\frac{1}{ik_0 r}-\frac{1}{k_0^2 r^2}\right)\int_S \left(|\boldsymbol{p}|^2 - |p_r|^2\right) ds \quad \text{(S23)}$$

Next, integrating the obtained value over the surface *S* gives:

$$\int_S \left(\boldsymbol{E}_p \times \boldsymbol{H}_p^*\right). d\boldsymbol{s} = \left(\frac{c_0}{\varepsilon_0}\right)\left(\frac{k_0^3}{4\pi k_0 r}\right)^2\left(1-\frac{1}{ik_0 r}\right)\left(1+\frac{1}{ik_0 r}-\frac{1}{k_0^2 r^2}\right)\int_S \left(|\boldsymbol{p}|^2 - |p_r|^2\right) ds \quad \text{(S24)}$$

where $d\boldsymbol{s} = ds\,\hat{r}$ is the differential surface vector normal to *S*. Finally, using $\int_S \left(|\boldsymbol{p}|^2 - |p_r|^2\right) ds = 8\pi r^2 |\boldsymbol{p}|^2 / 3$, we obtain the real part of Equation S24 as:

$$\frac{1}{2}\mathrm{Re}\int_S \left(\boldsymbol{E}_p \times \boldsymbol{H}_p^*\right) \cdot d\boldsymbol{s} = \frac{\eta_0 c_0^2 k_0^4}{12\pi} |\boldsymbol{p}|^2 \quad \text{(S25)}$$

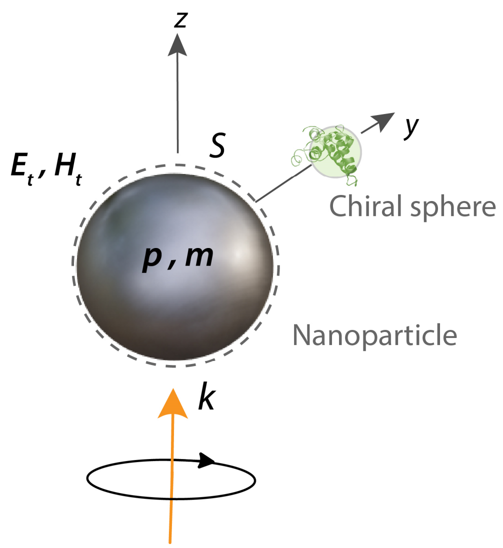

Figure S2. A chiral sphere near an achiral nanoparticle illuminated by a circularly polarized plane wave.

Similarly, we obtain:

$$\frac{1}{2}\mathrm{Re}\int_S \left(\boldsymbol{E}_m \times \boldsymbol{H}_m^*\right). d\boldsymbol{s} = \frac{\eta_0 k_0^4}{12\pi}|\boldsymbol{m}|^2 \quad \text{(S26)}$$

For $\boldsymbol{E}_{inc} \times \boldsymbol{H}_p^*$, we use the incident electric field in Equation S1 and the Green's function $\overleftrightarrow{\boldsymbol{G}}_{HP}$ in Equation S13, which gives the cross product:

$$\boldsymbol{E}_{inc} \times \boldsymbol{H}_p^* = c_0 k_0^3 \frac{\exp(ik_0 r)}{4\pi k_0 r}\left(1 - \frac{1}{ik_0 r}\right)\exp(-ik_0 z)\, \boldsymbol{E}_0 \times \left(\hat{r} \times \boldsymbol{p}^*\right) \quad \text{(S27)}$$

where $\boldsymbol{E}_0$ is the incident electric field vector at the center of the coordinate system. By integrating over the surface S and using $\boldsymbol{E}_0 \times \left(\hat{r} \times \boldsymbol{p}^*\right) = \left(\boldsymbol{E}_0 \cdot \boldsymbol{p}^*\right)\hat{r} - \left(\boldsymbol{E}_0 \cdot \hat{r}\right) \boldsymbol{p}^*$, Equation S27 can be rewritten as:

$$\int_S \left(\boldsymbol{E}_{inc} \times \boldsymbol{H}_p^*\right) \cdot d\boldsymbol{s} = c_0 k_0^3 \frac{\exp(ik_0 r)}{4\pi k_0 r}\left(1 - \frac{1}{ik_0 r}\right)\int_S \exp(-ik_0 r\cos\theta)\left(\boldsymbol{E}_0 \cdot \boldsymbol{p}^* - E_{0r}\, p_r^*\right) ds \quad \text{(S28)}$$

To calculate Equation S28, we first compute the integrals $\int_S \exp(-ik_0 r\cos\theta)\, \left(\boldsymbol{E}_0 \cdot \boldsymbol{p}^*\right) ds$ and $\int_S \exp(-ik_0 r\cos\theta)\, \left(E_{0r}\, p_r^*\right)\, ds$. The first one is simply given as:

$$\int_S \exp(-ik_0 r\cos\theta)\left(\boldsymbol{E}_0 \cdot \boldsymbol{p}^*\right) ds = \left(E_{0x} p_x^* + E_{0y} p_y^* + E_{0z} p_z^*\right) 4\pi r^2 \frac{\sin k_0 r}{k_0 r} \quad \text{(S29)}$$

where the equation $\int_S \exp(-ik_0 r\cos\theta)\, ds = 4\pi r^2 \sin k_0 r / k_0 r$ is used, and the x, y, and z subscripts indicate the components of the incident electric field or the electric dipole moments. The second integral form is:

$$\int_S \exp(-ik_0 r\cos\theta)\left(E_{0r}\, p_r^*\right)ds =$$
$$\pi r^2\left(E_{0x}p_x^* + E_{0y}p_y^*\right)\left(\frac{4\sin k_0 r - 4k_0 r\cos k_0 r}{k_0^3 r^3}\right) + 2\pi r^2 E_{0z}p_z^*\left(\frac{2\left(k_0^2 r^2 - 2\right)\sin k_0 r + 4k_0 r\cos k_0 r}{k_0^3 r^3}\right) \quad \text{(S30)}$$

for which we have used the identities $\int_S \exp(-ik_0 r\cos\theta)\sin^3\theta\, d\theta = \left(4\sin k_0 r - 4k_0 r\cos k_0 r\right)/k_0^3 r^3$ and $\int_S \exp(-ik_0 r\cos\theta)\cos^2\theta\,\sin\theta\, d\theta = \left[2\left(k_0^2 r^2 - 2\right)\sin k_0 r + 4k_0 r\cos k_0 r\right]/k_0^3 r^3$. Putting these integral values in Equation S28 results in:

$$\int_S \left(\boldsymbol{E}_{inc}\times\boldsymbol{H}_p^*\right)\cdot d\boldsymbol{s} = c_0 k_0^3\frac{\exp(ik_0 r)}{4\pi k_0 r}\left(1-\frac{1}{ik_0 r}\right)\left(4\pi r^2\right)$$
$$\left\{\left(\boldsymbol{E}_0\cdot\boldsymbol{p}^*\right)\left(\frac{\sin k_0 r}{k_0 r} - \frac{\sin k_0 r}{k_0^3 r^3} + \frac{\cos k_0 r}{k_0^2 r^2}\right) - E_{0z}p_z^*\left(\frac{\sin k_0 r}{k_0 r} - \frac{3\sin k_0 r}{k_0^3 r^3} + \frac{3\cos k_0 r}{k_0^2 r^2}\right)\right\} \quad \text{(S31)}$$

Likewise, we can obtain:

$$\int_S \left(\boldsymbol{E}_p\times\boldsymbol{H}_{inc}^*\right)\cdot d\boldsymbol{s} =$$
$$c_0 k_0^3\frac{\exp(ik_0 r)}{4\pi k_0 r}\left(4\pi r^2\right)\left(1-\frac{1}{ik_0 r}-\frac{1}{k_0^2 r^2}\right)\left(\frac{i\left(k_0 r\cos k_0 r - \sin k_0 r\right)}{k_0^2 r^2}\right)\left(\boldsymbol{E}_0\cdot\boldsymbol{p}^* - E_{0z}p_z^*\right) \quad \text{(S32)}$$

assuming that the induced dipole moments are in the *xy*-plane. Adding the two expressions in Equations S31 and S32 simplifies the results, yielding:

$$\frac{1}{2}\mathrm{Re}\int_S \left(\boldsymbol{E}_{inc}\times\boldsymbol{H}_p^* + \boldsymbol{E}_p\times\boldsymbol{H}_{inc}^*\right)\cdot d\boldsymbol{s} = -\frac{\omega_0}{2}\mathrm{Im}\left(\boldsymbol{E}_0\cdot\boldsymbol{p}^*\right) \quad \text{(S33)}$$

which represents the interaction of the incident electric field and the electric dipole. Through a similar approach, the interaction of the incident magnetic field and the magnetic dipole gives:

$$\frac{1}{2}\mathrm{Re}\int_S \left(\boldsymbol{E}_{inc}\times\boldsymbol{H}_m^* + \boldsymbol{E}_m\times\boldsymbol{H}_{inc}^*\right)\cdot d\boldsymbol{s} = -\frac{\omega_0}{2}\mathrm{Im}\left(\boldsymbol{B}_0\cdot\boldsymbol{m}^*\right) \quad \text{(S34)}$$

It is straightforward to show that the integration value for three other terms is zero:

$$\mathrm{Re}\int_S \left(\boldsymbol{E}_{inc}\times\boldsymbol{H}_{inc}^*\right)\cdot d\boldsymbol{s} = \mathrm{Re}\int_S \left(\boldsymbol{E}_p\times\boldsymbol{H}_m^*\right)\cdot d\boldsymbol{s} = \mathrm{Re}\int_S \left(\boldsymbol{E}_m\times\boldsymbol{H}_p^*\right)\cdot d\boldsymbol{s} = 0 \quad \text{(S35)}$$

By substituting Equations S33, S34, and S35 into Equation S21, we obtain the absorption inside the nanoparticle as:

$$P_{abs} = P_{ext} - P_{sca} \quad \text{(S36)}$$

where P_{ext} and P_{sca} are the extinct and scattered powers of the nanoparticle, which are expressed as:

$$P_{ext} = \frac{\omega_0}{2}\mathrm{Im}\left(\boldsymbol{E}_0\cdot\boldsymbol{p}^* + \boldsymbol{B}_0\cdot\boldsymbol{m}^*\right) \quad \text{(S37)}$$

and,

$$P_{sca} = \frac{\eta_0 k_0^4}{12\pi}\left(c_0^2 \left|\boldsymbol{p}\right|^2 + \left|\boldsymbol{m}\right|^2\right) \tag{S38}$$

respectively. Finally, recalling $\boldsymbol{p} = \alpha_e^i \boldsymbol{E}^i$ and $\boldsymbol{m} = \alpha_m^i \boldsymbol{H}^i$ relations (Equation S2), we can express the difference in extinct (ΔP_{ext}) and scattered (ΔP_{sca}) powers for the right- and left-handed circularly polarized excitations as:

$$\Delta P_{ext} = -\frac{\omega_0}{2}\operatorname{Im}\left(\alpha_e^i\right)\Delta\left|\boldsymbol{E}^i\right|^2 - \frac{\omega_0}{2}\mu_0 \operatorname{Im}\left(\alpha_m^i\right)\Delta\left|\boldsymbol{H}^i\right|^2 \tag{S39}$$

and,

$$\Delta P_{sca} = \frac{\eta_0 k_0^4 c_0^2}{12\pi}\left|\alpha_e^i\right|^2 \Delta\left|\boldsymbol{E}^i\right|^2 + \frac{\eta_0 k_0^4}{12\pi}\left|\alpha_m^i\right|^2 \Delta\left|\boldsymbol{H}^i\right|^2 \tag{S40}$$

respectively. $\Delta\left|\boldsymbol{E}^i\right|^2$ and $\Delta\left|\boldsymbol{H}^i\right|^2$ are the change in the intensity of the local electric and magnetic fields at the center of the nanoparticle for the right- and the left-handed circularly polarized excitations, which turn out to be non-zero in the presence of the chiral sphere.

Now, we examine these terms (Equations S37 and S38 or Equations S39 and S40) for the system shown in Figure S2 and for a plasmonic and a dielectric nanosphere (Figure S3). For each right- and left-circularly polarized excitation, we obtain the induced dipole moments in the nanosphere using the self-consistent systems of Equations derived in Supporting Section S1 (Equations S16 and S17). The radius of the chiral sphere is 5 nm, and it is placed 5 nm away from the surface of the nanoparticle, while the radius of the nanoparticle varies between 25 and 100 nm. To get more tangible numbers, we normalize all values to the differential absorption of the chiral sphere in free space, which is expressed as $\Delta P_{0c} = -4\omega E_0^2 \operatorname{Re}\left(\alpha_{em}^c\right)/\eta_0$, where ω is the angular frequency and α_{em}^c is the cross-polarizability of the chiral sphere (Equation S3). We see that the presence of the chiral sphere gives rise to non-zero differential absorption/scattering/extinction in both plasmonic and dielectric systems. This is due to the different magnitudes of the local fields for the right- and left-handed circularly polarized excitations ($\mathbf{E}_R^i \neq \mathbf{E}_L^i$, $\mathbf{H}_R^i \neq \mathbf{H}_L^i$), which effectively makes the achiral nanoparticle act like a chiral particle. Therefore, the phenomenon can be dubbed *chirality transfer*. We also see that the chirality transfer to the dielectric nanosphere is considerably higher than that of plasmonic nanoparticle (compare Figure S3a-S3c to Figure S3d-S3e), which drives us toward exploiting dielectric platforms for chiral detection enabled by chirality transfer. Furthermore, for this dielectric nanoparticle, we see that the differential absorption is maximized at a specific radius (Figure S3a) which highlights the importance of the rational design of such nanostructures for maximized chiral signal.

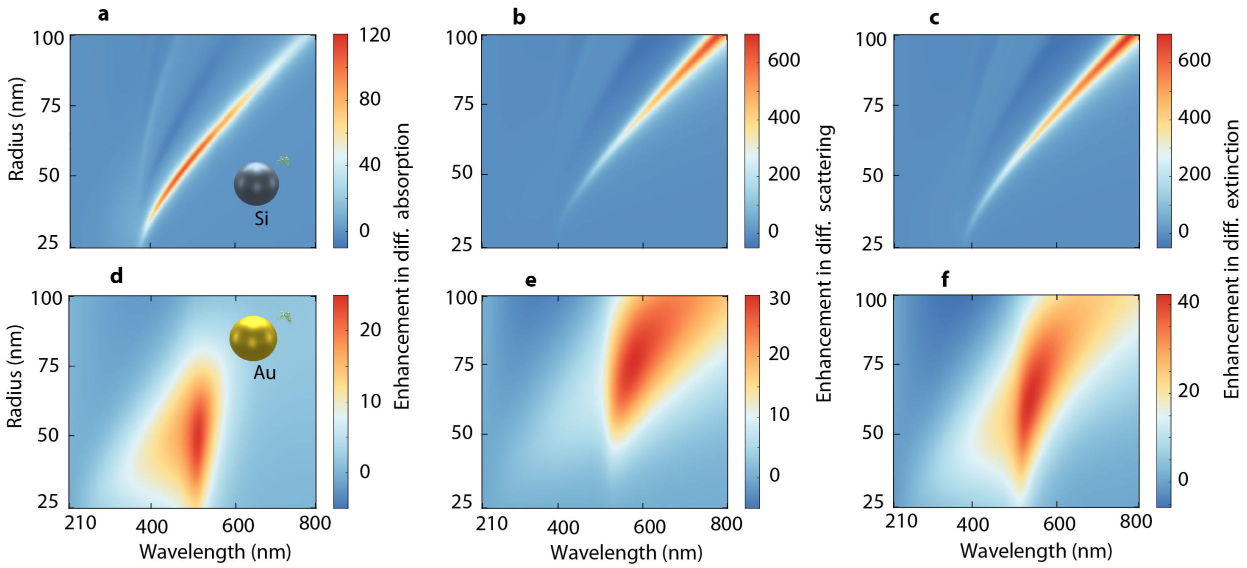

Figure S3. Enhancement in differential absorption, (a), (d), differential scattering (b), (e) and differential extinction (c), (f) for a dielectric (a)-(c) and a plasmonic (c)-(f) nanoparticle, due to chirality transfer. The system under study is shown in Figure S2, with a radius of the chiral sphere of 5 nm placed 5 nm away (surface-to-surface) from the nanoparticle. The Pasteur parameter and the permittivity for the chiral sphere are taken from the calibrated Lorentzian in Equations S8 and S9 (see also Supporting Section S7). The dielectric and the plasmonic nanoparticles are made of silicon and gold with realistic permittivity.[7,8] The absorbed, extinct, and scattered powers for right- and left-handed circularly polarized excitations are calculated using Equations S36, S37, and S38, respectively.

Next, we decompose Equations S39 and S40 in terms of electric and magnetic parts as:

$$\Delta P_{ext,E} = -\frac{\omega_0}{2}\operatorname{Im}\left(\alpha_e^i\right)\Delta\left|\boldsymbol{E}^i\right|^2 \tag{S41}$$

$$\Delta P_{ext,H} = -\frac{\omega_0}{2}\mu_0\operatorname{Im}\left(\alpha_m^i\right)\Delta\left|\boldsymbol{H}^i\right|^2 \tag{S42}$$

$$\Delta P_{sca,E} = \frac{\eta_0 k_0^4 c_0^2}{12\pi}\left|\alpha_e^i\right|^2\Delta\left|\boldsymbol{E}^i\right|^2 \tag{S43}$$

$$\Delta P_{sca,H} = \frac{\eta_0 k_0^4 c_0^2}{12\pi}\left|\alpha_m^i\right|^2\Delta\left|\boldsymbol{H}^i\right|^2 \tag{S44}$$

where $\Delta P_{ext,E}$ and $\Delta P_{ext,H}$ ($\Delta P_{sca,E}$ and $\Delta P_{sca,H}$) are the electric and magnetic chirality transfer observed in the differential extinct (differential scattered) power. Physically, the electric (magnetic) chirality transfer is due to a small difference between the electric (magnetic) energy density at the position of the nanoparticle upon excitation by right- and left-circularly polarized light. Figure S4 shows the normalized electric and magnetic chirality transfer in extinct power (Equation S41 and S42) for a dielectric and a plasmonic nanosphere. To calculate $\Delta\left|\boldsymbol{E}^i\right|^2$ and $\Delta\left|\boldsymbol{H}^i\right|^2$, we first compute the dipole moments using the self-consistent system of Equations S16 and S17. Then, we obtain the local fields at the location of the nanoparticle using dyadic Green's functions in Equations S12-S15. We see that for the dielectric particle

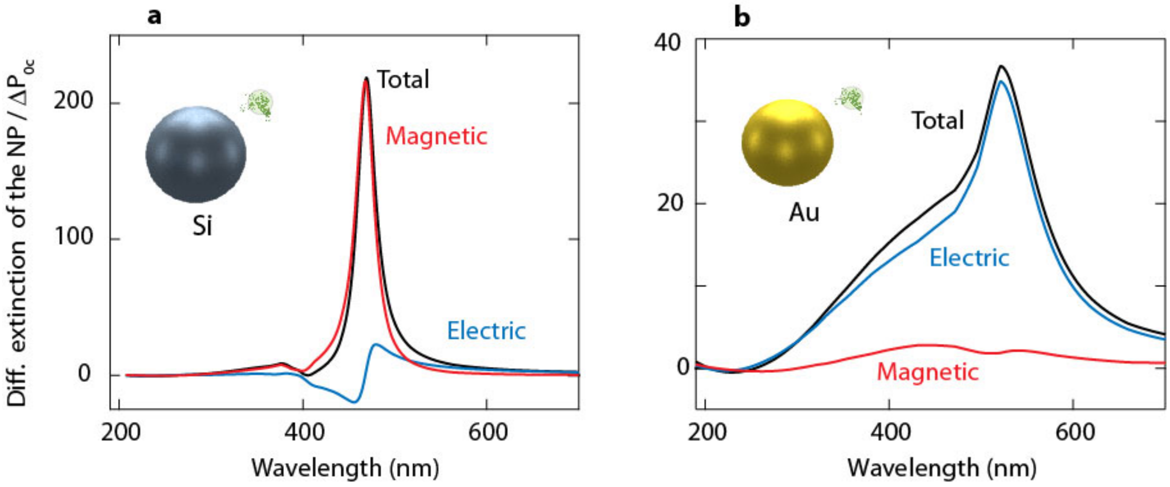

Figure S4. Electric and magnetic contributions to the differential extinction of (a) a dielectric (silicon) and (b) a plasmonic (gold) nanoparticle of radius 50 nm. The chiral sphere has a radius of 5 nm, and it is placed 5 nm away (surface-to-surface) from the NP (see Figure S2). This configuration is investigated using the self-consistent system of equations derived in Supporting Section S1 (Equations S16 and S17).

(Figure S4a) the dominant part of the total chirality transfer (black curve) is magnetic (red curve), while for the plasmonic one (Figure S4b), the electric contribution (blue curve) is dominant.

Supporting Section S3. Transfer of chirality from a small chiral sphere to an achiral nanoparticle

In this Supporting Section, we present a theoretical description of chirality transfer in the dipolar regime. To do this, starting from Equations S39 and S40, we express the differential extinction ($\Delta P^i_{ext} = P^i_{ext,R} - P^i_{ext,L}$) and the differential scattering ($\Delta P^i_{sca} = P^i_{sca,R} - P^i_{sca,L}$) of the nanoparticle as:

$$\Delta P^i_{ext} = -\frac{\omega_0}{2}\left(\mathrm{Im}\left(\alpha^i_e\right)\Delta\left|\boldsymbol{E}^i\right|^2 + \mu_0\,\mathrm{Im}\left(\alpha^i_m\right)\Delta\left|\boldsymbol{H}^i\right|^2\right) \quad \text{(S45)}$$

$$\Delta P^i_{sca} = \frac{\eta_0 k_0^4}{12\pi}\left(c_0{}^2\left|\alpha^i_e\right|^2\Delta\left|\boldsymbol{E}^i\right|^2 + \left|\alpha^i_m\right|^2\Delta\left|\boldsymbol{H}^i\right|^2\right) \quad \text{(S46)}$$

respectively, where $\Delta\left|\boldsymbol{E}^i\right|^2 = \left|\boldsymbol{E}^i_R\right|^2 - \left|\boldsymbol{E}^i_L\right|^2$ and $\Delta\left|\boldsymbol{H}^i\right|^2 = \left|\boldsymbol{H}^i_R\right|^2 - \left|\boldsymbol{H}^i_L\right|^2$ account for the differential change of the electric and magnetic fields due to the presence of the chiral sphere, and are expressed as:

$$\Delta\left|\boldsymbol{E}^i\right|^2 = \Delta\left|E^i_x\right|^2 + \Delta\left|E^i_y\right|^2 + \Delta\left|E^i_z\right|^2 \quad \text{(S47)}$$

$$\Delta\left|\boldsymbol{H}^i\right|^2 = \Delta\left|H^i_x\right|^2 + \Delta\left|H^i_y\right|^2 + \Delta\left|H^i_z\right|^2 \quad \text{(S48)}$$

where $\Delta\left|E^i_\alpha\right|^2 = \left|E^i_{\alpha,R}\right|^2 - \left|E^i_{\alpha,L}\right|^2$. On the other hand, the electric and magnetic field components at the position of the nanoparticle are (Equation S12-S15):

$$
\begin{aligned}
E^{i}_{x,R/L} &= E_0 + \left(k_0^3/\varepsilon_0\right)\rho\left(k_0 l\right)p^c_x + \eta_0 k_0^3\ \tau\left(k_0 l\right)m^c_z \\
E^{i}_{y,R/L} &= \mp\, iE_0 + \left(k_0^3/\varepsilon_0\right)\zeta\left(k_0 l\right)p^c_y \\
E^{i}_{z,R/L} &= \left(k_0^3/\varepsilon_0\right)\rho\left(k_0 l\right)p^c_z - \eta_0 k_0^3\ \tau\left(k_0 l\right)m^c_x
\end{aligned}
\tag{S49}
$$

and,

$$
\begin{aligned}
H^{i}_{x,R/L} &= \pm iE_0/\eta_0 - c_0 k_0^3\ \tau\left(k_0 l\right)p^c_z + k_0^3\rho\left(k_0 l\right)m^c_x \\
H^{i}_{y,R/L} &= E_0/\eta_0 + k_0^3\zeta\left(k_0 l\right)m^c_y \\
H^{i}_{z,R/L} &= c_0 k_0^3\ \tau\left(k_0 l\right)p^c_x + k_0^3\rho\left(k_0 l\right)m^c_z
\end{aligned}
\tag{S50}
$$

respectively, where the upper (lower) sign in "$\pm/\mp$" is used for the right- (left-)handed circularly polarized excitation (see Equation S1). To obtain the differential extinction and scattering in Equations S45 and S46, we must obtain all six components in Equations S47 and S48. For brevity, here we only show the full derivation of $\Delta\left|E^i_x\right|^2$ and $\Delta\left|E^i_y\right|^2$. It is straightforward to use the same approach for the other components. Given that the chiral sphere is very small, we assume that it does not change the near field of the NP. Thus, we can approximate the local fields at the location of the chiral sphere as the near fields of the nanoparticle when excited alone without the presence of the chiral sphere. This gives the dipole moments of the chiral sphere in Equations S3 in terms of such near fields, which in turn eventually provides the local fields at the location of the nanoparticle in terms of its own near fields. For example, the *x*- and *y*-components of the local electric field at the position of the NP can be written as:

$$
E^{i}_{x,R/L} = E_0 + \left(k_0^3/\varepsilon_0\right)\rho\left(k_0 l\right)\left(\alpha^c_{ee}E^c_{x,R/L} + \alpha^c_{em}H^c_{x,R/L}\right) + \eta_0 k_0^3\ \tau\left(k_0 l\right)\left(\alpha^c_{me}E^c_{z,R/L} + \alpha^c_{mm}H^c_{z,R/L}\right) \tag{S51}
$$

$$
E^{i}_{y,R/L} = \mp iE_0 + \left(k_0^3/\varepsilon_0\right)\zeta\left(k_0 l\right)\left(\alpha^c_{ee}E^c_{y,R/L} + \alpha^c_{em}H^c_{y,R/L}\right) \tag{S52}
$$

where $E^c_{\alpha,R/L}$ ($H^c_{\alpha,R/L}$) is the α -component of the near electric (magnetic) field at the location of the chiral sphere for right-(R) or left-handed (L) circularly polarized excitation.

From Equation S51 and taking the symmetry conditions $E^c_{x,L} = E^c_{x,R}$, $H^c_{x,L} = -H^c_{x,R}$, $E^c_{z,L} = -E^c_{z,R}$, and $H^c_{z,L} = H^c_{z,R}$ into account, we can approximate $\Delta\left|E^i_x\right|^2$ as:

$$
\left|E^i_{x,R}\right|^2 - \left|E^i_{x,L}\right|^2 \simeq \frac{4k_0^3E_0}{\varepsilon_0}\mathrm{Re}\left[\alpha^c_{em}\left(\rho\left(k_0 l\right)\mathrm{H}^c_{x,R} - \frac{1}{\eta_0}\ \tau\left(k_0 l\right)\mathrm{E}^c_{z,R}\right)\right] \tag{S53}
$$

Similarly, from Equation S52 and using the symmetry conditions $E^c_{y,L} = -E^c_{y,R}$ and $H^c_{y,L} = H^c_{y,R}$, we can calculate $\Delta\left|E^i_y\right|^2$ as:

$$
\left|E^i_{y,R}\right|^2 - \left|E^i_{y,L}\right|^2 \simeq \frac{4k_0^3E_0}{\varepsilon_0}\mathrm{Re}\left(i\,\zeta\left(k_0 l\right)\alpha^c_{em}\mathrm{H}^c_{y,R}\right) \tag{S54}
$$

Through the self-consistent solution, it can be shown that the contribution from $\Delta\left|E^i_z\right|^2$ is negligible compared to $\Delta\left|E^i_x\right|^2$ and $\Delta\left|E^i_y\right|^2$. Therefore, adding Equations S53 and S54, $\Delta\left|\boldsymbol{E}^i\right|^2$ can be obtained as:

$$\Delta\left|\boldsymbol{E}^{i}\right|^{2}\simeq\frac{4E_{0}k_{0}^{3}}{\varepsilon_{0}}\mathrm{Re}\left[\alpha_{em}^{c}\left(\rho(k_{0}l)H_{x,R}^{c}-\frac{1}{\eta_{0}}\tau(k_{0}l)E_{z,R}^{c}+i\zeta(k_{0}l)H_{y,R}^{c}\right)\right] \tag{S55}$$

Analogously, $\Delta\left|\boldsymbol{H}^{i}\right|^{2}$ can be derived as:

$$\Delta\left|\boldsymbol{H}^{i}\right|^{2}=\frac{4E_{0}k_{0}^{3}}{\eta_{0}}\frac{1}{\mu_{0}}\mathrm{Re}\left[i\alpha_{em}^{c}\left(\rho(k_{0}l)E_{x,R}^{c}+\eta_{0}\tau(k_{0}l)H_{z,R}^{c}+i\zeta(k_{0}l)E_{y,R}^{c}\right)\right] \tag{S56}$$

Substituting Equations S55 and S56 into Equations S45 and S46 gives the differential extinct and scattered powers as:

$$\Delta P_{ext}^{i}=12\pi E_{0}\omega_{0}\left(\begin{array}{l}\mathrm{Re}(a_{1})\mathrm{Re}\left[\alpha_{em}^{c}\left(\rho(k_{0}l)H_{x,R}^{c}-\frac{1}{\eta_{0}}\tau(k_{0}l)E_{z,R}^{c}+i\zeta(k_{0}l)H_{y,R}^{c}\right)\right]+\\ \mathrm{Re}(b_{1})\mathrm{Re}\left[i\alpha_{em}^{c}\left(\frac{1}{\eta_{0}}\rho(k_{0}l)E_{x,R}^{c}+\tau(k_{0}l)H_{z,R}^{c}+\frac{i}{\eta_{0}}\zeta(k_{0}l)E_{y,R}^{c}\right)\right]\end{array}\right) \tag{S57}$$

$$\Delta P_{sca}^{i}=12\pi E_{0}\omega_{0}\left(\begin{array}{l}|a_{1}|^{2}\,\mathrm{Re}\left[\alpha_{em}^{c}\left(\rho(k_{0}l)H_{x,R}^{c}-\frac{1}{\eta_{0}}\tau(k_{0}l)E_{z,R}^{c}+i\zeta(k_{0}l)H_{y,R}^{c}\right)\right]+\\ |b_{1}|^{2}\,\mathrm{Re}\left[i\alpha_{em}^{c}\left(\frac{1}{\eta_{0}}\rho(k_{0}l)E_{x,R}^{c}+\tau(k_{0}l)H_{z,R}^{c}+\frac{i}{\eta_{0}}\zeta(k_{0}l)E_{y,R}^{c}\right)\right]\end{array}\right) \tag{S58}$$

where we have converted the polarizabilities of the nanoparticle to the corresponding Mie coefficients through $\alpha_{e}^{i}=\left(\frac{-i\,6\pi\varepsilon_{0}}{k_{0}^{3}}a_{1}\right)$ and $\alpha_{m}^{i}=\left(\frac{-i\,6\pi}{k_{0}^{3}}b_{1}\right)$.[2] Figure S5 shows a comparison between the differential extinct power of the NP obtained through the self-consistent system (Equations S16 and S17, and Equation 39) and close-form analytical derivation in Equation S57. We see a perfect agreement between the two methods, which shows the validity of approximations we made to derive Equation S57.

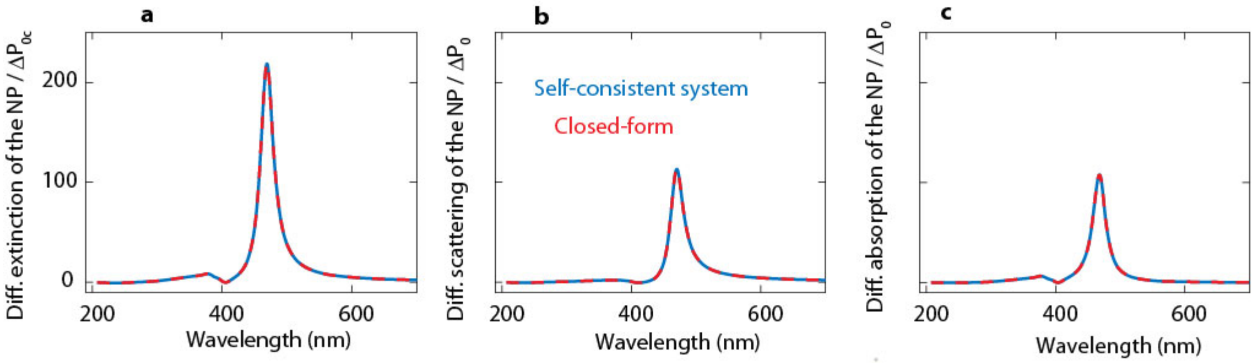

Figure S5. The comparison between the solutions from the self-consistent system of Equation S16, S17 (blue) and the analytical closed-form expressions in Equation S57, for which all dipole components are considered. (a), (b), and (c) represent the differential extinct power, the differential scattered power, and the differential absorbed power for a silicon nanoparticle of radius 50 nm. The chiral sphere has a radius of 5 nm, and it is placed 5 nm away (surface-to-surface) from the NP. The permittivity and the Pasteur parameter for the chiral sphere come from the calibrated Lorentzian model in Equations S8 and S9 (see also Supporting Section S7).

In dimer systems like the one in Figure S1, the dominant dipole moments are those along the dimer axis[9] (*i.e.*, p_y^c, m_y^c for the chiral sphere), which generate the fields through the $\zeta(k_0 l)$ function (see Equations S16 and S17). To further simplify the results, we can consider only the contributions of these dominant dipoles in Equations S57 and S58, which gives:

$$\frac{\Delta P_{ext}^i}{\Delta P_{0c}} \simeq \frac{-3\pi}{\mathrm{Re}(\alpha_{em}^c)}\left(\mathrm{Re}(a_1)\mathrm{Re}\left(i\,\alpha_{em}^c \zeta(k_0 l)\frac{H_{y,R}^c}{H_0}\right) - \mathrm{Re}(b_1)\mathrm{Re}\left(\alpha_{em}^c \zeta(k_0 l)\frac{E_{y,R}^c}{E_0}\right)\right) \quad \text{(S59)}$$

$$\frac{\Delta P_{sca}^i}{\Delta P_{0c}} \simeq \frac{-3\pi}{\mathrm{Re}(\alpha_{em}^c)}\left(|a_1|^2\,\mathrm{Re}\left(i\,\alpha_{em}^c \zeta(k_0 l)\frac{H_{y,R}^c}{H_0}\right) - |b_1|^2\,\mathrm{Re}\left(\alpha_{em}^c \zeta(k_0 l)\frac{E_{y,R}^c}{E_0}\right)\right) \quad \text{(S60)}$$

where $H_0 = E_0/\eta_0$ and ΔP_0 is the differential extinct power of the chiral sphere in free space given by $\Delta P_{0c} = -4\omega_0 E_0^2\,\mathrm{Re}(\alpha_{em}^c)/\eta_0$. From Equations S59 and S60, we see that the electric and the magnetic parts of the chirality transfer (the first and the second terms on the right-hand side of Equations) are related to the magnetic ($H_{y,R}^c/H_0$) and the electric ($E_{y,R}^c/E_0$) field enhancement at the location of the chiral sphere. In fact, magnetic and electric field enhancement act on the chiral sphere, and their reaction toward the NP gives rise to the differential change in the electric ($\Delta|\boldsymbol{E}^i|^2$; Equation S55) and magnetic field intensity ($\Delta|\boldsymbol{H}^i|^2$; Equation S56) at the position of the NP.

Finally, by using the tailor expansion of the $\zeta(k_0 l)$ function in Equations S59 and S60, the differential extinct and scattered powers can be obtained as:

$$\frac{\Delta P_{ext}^i}{\Delta P_0} = \frac{-3}{2\,\mathrm{Re}(\alpha_{em}^c)}\left(\frac{1}{k_0^3 l^3} + \frac{1}{2k_0 l}\right)\left[\mathrm{Re}(a_1)\mathrm{Im}\left(\alpha_{em}^c H_{y,R}^{c,\,enh}\right) + \mathrm{Re}(b_1)\mathrm{Im}\left(\alpha_{em}^c\ E_{y,R}^{c,\,enh}\right)\right] \quad \text{(S61)}$$

$$\frac{\Delta P_{sca}^i}{\Delta P_0} = \frac{-3}{2\,\mathrm{Re}(\alpha_{em})}\left(\frac{1}{k_0^3 l^3} + \frac{1}{2k_0 l}\right)\left(|a_1|^2\,\mathrm{Im}\left(\alpha_{em}^c H_{y,R}^{c,\,enh}\right) + |b_1|^2\,\mathrm{Im}\left(\alpha_{em}^c\ E_{y,R}^{c,\,enh}\right)\right) \quad \text{(S62)}$$

where $E_{y,R}^{c,\,enh} = E_{y,R}^c/(-iE_0)$ and $H_{y,R}^{c,\,enh} = H_{y,R}^c/H_0$.

In Figure S6, we plot these equations for the same system in Figure S5 (red curves) and compare the results to the direct solution from the self-consistent system (blue curves). We see a good agreement between the two methods, which shows the validity of the dominant dipole moment approximation.

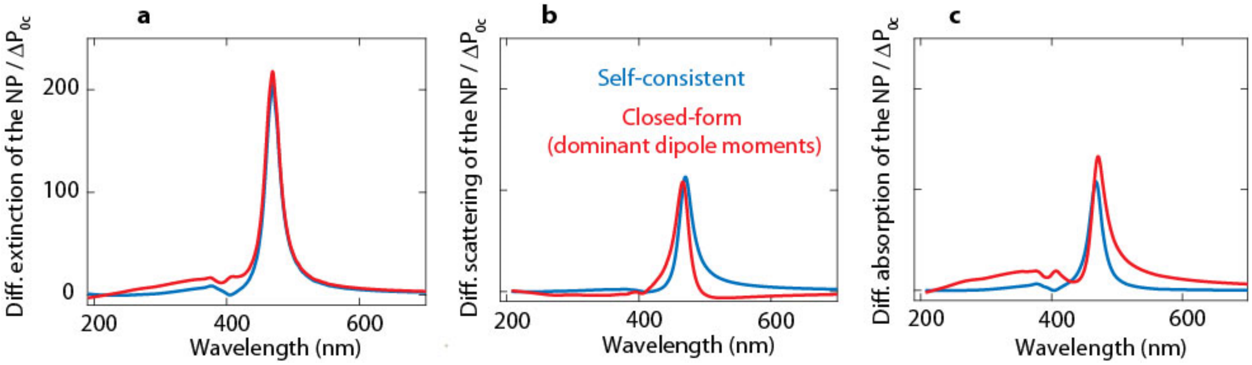

Figure S6. Comparison between the solutions from the self-consistent system of Equations S16 and S17 (blue) and the analytical closed-form expressions in Equations S61 and S62, for which only y-components of the dipole moments are considered. (a), (b), and (c) represent the differential extinct power (b), the differential scattered power, and (b) the differential absorbed power for a silicon nanoparticle of a radius of 50 nm. The chiral sphere has a radius of 5 nm, and it is placed 5 nm away (surface-to-surface) from the NP. The permittivity and the Pasteur parameter for the chiral sphere come from the calibrated Lorentzian model in Equations S8 and S9 (see also Supporting Section S7).

According to Equation S5, the real and the imaginary parts of the cross-polarizability α_{em}^{c} are proportional to $\mathrm{Im}(\kappa)$ and $-\mathrm{Re}(\kappa)$, respectively. Thus, we can rewrite Equations S61 and S62 in terms of the Pasteur parameter as:

$$\frac{\Delta P_{ext}^{i}}{\Delta P_{0c}} = \frac{-3}{2\,\mathrm{Im}(\kappa)}\left(\frac{1}{k_0^3 l^3}+\frac{1}{2k_0 l}\right)\left[\mathrm{Re}(a_1)\mathrm{Re}\left(\kappa H_{y,R}^{c,enh}\right)+\mathrm{Re}(b_1)\mathrm{Re}\left(\kappa\, E_{y,R}^{c,enh}\right)\right] \quad \text{(S63)}$$

$$\frac{\Delta P_{sca}^{i}}{\Delta P_{0c}} = \frac{-3}{2\,\mathrm{Im}(\kappa)}\left(\frac{1}{k_0^3 l^3}+\frac{1}{2k_0 l}\right)\left(|a_1|^2\,\mathrm{Re}\left(\kappa\, H_{y,R}^{c,enh}\right)+|b_1|^2\,\mathrm{Re}\left(\kappa\, E_{y,R}^{c,enh}\right)\right) \quad \text{(S64)}$$

Supporting Section S4. Transfer of chirality from a chiral shell to an achiral nanoparticle

Figure S7 shows an achiral resonant NP of radius R_i covered by a thin chiral shell of thickness δ_s. The whole system is illuminated by a linearly polarized plane wave with the electric field $\boldsymbol{E}_{inc}(\boldsymbol{r}) = \exp(-ik_0 z)\,\hat{x}$. Here, instead of separate calculations for right- and left-handed circularly polarized excitations, we obtain the cross-polarization conversion for linearly polarized illumination. Then, we relate this cross-polarization term to the differential change in the electric and magnetic field intensity upon illumination by right- and left-handed circularly polarized excitations, which is what we need for the differential extinction or scattering calculations (Equations S45 and S46).

Suppose we illuminate the shell-nanoparticle system shown in Figure S7 with a plane wave linearly polarized along x. In that case, the local electric and magnetic fields at the center of the nanoparticle can be expressed as:

$$\begin{aligned} \boldsymbol{E}_{x\text{-}p}(0) &\simeq E_0\,\hat{x} + E_c\,\hat{y} \\ \boldsymbol{H}_{x\text{-}p}(0) &\simeq -H_c\,\hat{x} + H_0\,\hat{y} \end{aligned} \quad \text{(S65)}$$

where E_0 and $H_0 = E_0 / \eta_0$ are the amplitudes of the incident electric and magnetic fields, which remain almost unchanged because they are significantly larger than the small perturbations due to the chiral shell. On the other hand, E_c and H_c are the electric and magnetic fields (of small amplitude compared to the incident field) that capture the chirality transfer effects of the shell in the form of cross-conversion polarization. Due to symmetry, we can expect that illuminating the same system with a y-polarized plane wave produces the local fields:

$$\begin{aligned} \boldsymbol{E}_{y\text{-}p}(0) &\simeq -E_c\,\hat{x} + E_0\,\hat{y} \\ \boldsymbol{H}_{y\text{-}p}(0) &\simeq -H_0\,\hat{x} - H_c\,\hat{y} \end{aligned} \tag{S66}$$

Noting that the circular polarization is a superposition of two linear polarizations, we can build the local electric fields for right- and left-handed circularly polarized incident waves as:

$$\begin{aligned} \boldsymbol{E}_{R\text{-}p}(0) &= \left(E_0 + iE_c\right)\hat{x} + \left(-iE_0 + E_c\right)\hat{y} \\ \boldsymbol{E}_{L\text{-}p}(0) &= \left(E_0 - iE_c\right)\hat{x} + \left(iE_0 + E_c\right)\hat{y} \end{aligned} \tag{S67}$$

respectively, and the local magnetic fields as:

$$\begin{aligned} \boldsymbol{H}_{R\text{-}p}(0) &= \left(iH_0 - H_c\right)\hat{x} + \left(H_0 + iH_c\right)\hat{y} \\ \boldsymbol{H}_{L\text{-}p}(0) &= \left(-iH_0 - H_c\right)\hat{x} + \left(H_0 - iH_c\right)\hat{y} \end{aligned} \tag{S68}$$

respectively. From Equations S67 and S68, we can obtain the differential change in local electric and magnetic field intensity for RCP and LCP illuminations as:

$$\left|\boldsymbol{E}_{R-p}(0)\right|^2 - \left|\boldsymbol{E}_{L-p}(0)\right|^2 = 8\,\mathrm{Im}\left(E_0 E_c^*\right) \tag{S69}$$

$$\left|\boldsymbol{H}_{R-p}(0)\right|^2 - \left|\boldsymbol{H}_{L-p}(0)\right|^2 = 8\,\mathrm{Im}\left(H_0 H_c^*\right) \tag{S70}$$

respectively.

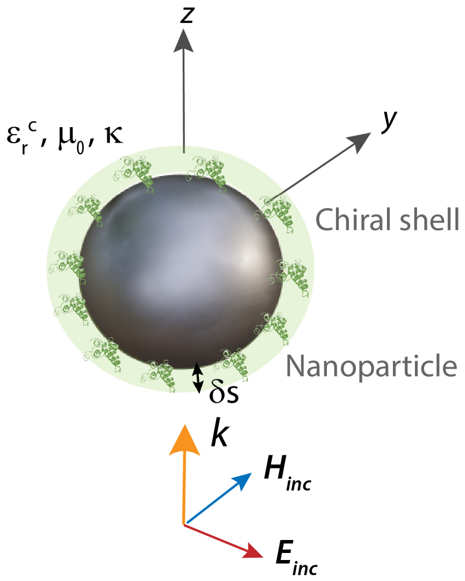

Figure S7. An achiral nanoparticle surrounded by a chiral shell of thickness δ_s illuminated by a circularly polarized plane wave.

I. Electric chirality transfer

The constitutive equations for an isotropic chiral medium are:

$$\begin{aligned} \boldsymbol{D} &= \varepsilon^c \boldsymbol{E} - i\frac{\kappa}{c_0}\boldsymbol{H} \\ \boldsymbol{B} &= \mu^c \boldsymbol{H} + i\frac{\kappa}{c_0}\boldsymbol{E} \end{aligned} \qquad \text{(S71)}$$

where ε^c and μ^c are the permittivity and the permeability and κ is the Pasteur parameter which denotes the chirality of the medium. From the $\boldsymbol{D} = \varepsilon_0\boldsymbol{E} + \boldsymbol{P}$ and $\boldsymbol{B} = \mu_0\left(\boldsymbol{H} + \boldsymbol{M}\right)$ relations, we can obtain the electric and the magnetic dipole densities for a non-magnetic ($\mu^c = \mu_0$) chiral medium as:

$$\boldsymbol{P} = \varepsilon_0\left(\varepsilon_r^c - 1\right)\boldsymbol{E} - i\frac{\kappa}{c_0}\boldsymbol{H} \qquad \text{(S72)}$$

$$\boldsymbol{M} = i\frac{\kappa}{\eta_0}\boldsymbol{E} \qquad \text{(S73)}$$

respectively, where ε_r^c is the relative permittivity. If we multiply both dipole densities by the differential volume of the chiral shell (dv), then each differential dipole acts like the chiral sphere in Supporting Section S1-S3. Then, their overall effect on the local electric field at the center of the NP is given by a direct integration over the volume of the shell as:

$$\boldsymbol{E}(0) = \boldsymbol{E}_{inc}(0) + \int_{shell} \overleftrightarrow{\boldsymbol{G}}_{EP}(0,\boldsymbol{r})\cdot\left[\varepsilon_0\left(\varepsilon_r^c - 1\right)\boldsymbol{E}\left(\boldsymbol{r}\right) - i\frac{\kappa}{c_0}\boldsymbol{H}\left(\boldsymbol{r}\right)\right]dv + \int_{shell} \overleftrightarrow{\boldsymbol{G}}_{EM}(0,\boldsymbol{r})\cdot\left[i\frac{\kappa}{\eta_0}\boldsymbol{E}\left(\boldsymbol{r}\right)\right]dv \qquad \text{(S74)}$$

where $\boldsymbol{E}\left(\boldsymbol{r}\right)$ and $\boldsymbol{H}\left(\boldsymbol{r}\right)$ are the total electric and total magnetic fields at the location of the chiral shell, which are expressed as:

$$\boldsymbol{E}\left(\boldsymbol{r}\right) = \boldsymbol{E}_{inc}(\boldsymbol{r}) + \alpha_e^i \overleftrightarrow{\boldsymbol{G}}_{EP}(\boldsymbol{r},0)\cdot\boldsymbol{E}_{inc}(0) + \alpha_m^i \overleftrightarrow{\boldsymbol{G}}_{EM}(\boldsymbol{r},0)\cdot\boldsymbol{H}_{inc}(0) \qquad \text{(S75)}$$

$$\boldsymbol{H}\left(\boldsymbol{r}\right) = \boldsymbol{H}_{inc}(\boldsymbol{r}) + \alpha_e^i \overleftrightarrow{\boldsymbol{G}}_{HP}(\boldsymbol{r},0)\cdot\boldsymbol{E}_{inc}(0) + \alpha_m^i \overleftrightarrow{\boldsymbol{G}}_{HM}(\boldsymbol{r},0)\cdot\boldsymbol{H}_{inc}(0) \qquad \text{(S76)}$$

respectively. Inserting these fields in Equation S74 yields the local electric field at the center of the NP in terms of the incident field and the Green's functions as:

$$\begin{aligned} &\boldsymbol{E}(0) = \boldsymbol{E}_{inc}(0) + \varepsilon_0\left(\varepsilon_r^c - 1\right)\int_{shell} \overleftrightarrow{\boldsymbol{G}}_{EP}(0,\boldsymbol{r})\cdot\boldsymbol{E}_{inc}(\boldsymbol{r})\,dv + \alpha_e^i\;\varepsilon_0\left(\varepsilon_r^c - 1\right)\int_{shell} \overleftrightarrow{\boldsymbol{G}}_{EP}(0,\boldsymbol{r})\cdot\overleftrightarrow{\boldsymbol{G}}_{EP}(\boldsymbol{r},0)\cdot\boldsymbol{E}_{inc}(0)\,dv + \\ &\alpha_m^i\;\varepsilon_0\left(\varepsilon_r^c - 1\right)\int_{shell} \overleftrightarrow{\boldsymbol{G}}_{EP}(0,\boldsymbol{r})\cdot\overleftrightarrow{\boldsymbol{G}}_{EM}(\boldsymbol{r},0)\cdot\boldsymbol{H}_{inc}(0)\,dv - i\frac{\kappa}{c_0}\int_{shell} \overleftrightarrow{\boldsymbol{G}}_{EP}(0,\boldsymbol{r})\cdot\boldsymbol{H}_{inc}(\boldsymbol{r})\,dv - \\ &i\alpha_e^i\;\frac{\kappa}{c_0}\int_{shell} \overleftrightarrow{\boldsymbol{G}}_{EP}(0,\boldsymbol{r})\cdot\overleftrightarrow{\boldsymbol{G}}_{HP}(\boldsymbol{r},0)\cdot\boldsymbol{E}_{inc}(0)\,dv - i\alpha_m^i\;\frac{\kappa}{c_0}\int_{shell} \overleftrightarrow{\boldsymbol{G}}_{EP}(0,\boldsymbol{r})\cdot\overleftrightarrow{\boldsymbol{G}}_{HM}(\boldsymbol{r},0)\cdot\boldsymbol{H}_{inc}(0)\,dv + \\ &i\frac{\kappa}{\eta_0}\int_{shell} \overleftrightarrow{\boldsymbol{G}}_{EM}(0,\boldsymbol{r})\cdot\boldsymbol{E}_{inc}(\boldsymbol{r})\,dv + i\alpha_e^i\;\frac{\kappa}{\eta_0}\int_{shell} \overleftrightarrow{\boldsymbol{G}}_{EM}(0,\boldsymbol{r})\cdot\overleftrightarrow{\boldsymbol{G}}_{EP}(\boldsymbol{r},0)\cdot\boldsymbol{E}_{inc}(0)\,dv + \\ &i\alpha_m^i\;\frac{\kappa}{\eta_0}\int_{shell} \overleftrightarrow{\boldsymbol{G}}_{EM}(0,\boldsymbol{r})\cdot\overleftrightarrow{\boldsymbol{G}}_{EM}(\boldsymbol{r},0)\cdot\boldsymbol{H}_{inc}(0)\,dv \end{aligned} \qquad \text{(S77)}$$

The most useful information from Equation S77 for chirality transfer is the y-component of the electric field. It can be shown that, among the nine terms in Equation S77, only four of them have non-zero y-components, for which we show the calculations next. The first one is $-i\left(\kappa / c_0\right) \int_{shell} \overleftrightarrow{\boldsymbol{G}}_{EP}(0,\boldsymbol{r}) \cdot \boldsymbol{H}_{inc}(\boldsymbol{r})\, dv$ in which the y-polarized incident magnetic field induces the y-components of the electric dipole moments on the shell. These electric dipole moments then generate the y-component of the local electric field as:

$$\hat{y}\ \left(-i\kappa k_0^3\right)\frac{\exp(-ik_0 r)}{4\pi k_0 r}\left(2\pi r^2\delta_s\right) \left\{\left(1+\frac{1}{ik_0 r}-\frac{1}{k_0^2 r^2}\right)\int_{shell}\exp\left(-ik_0 r\cos\theta\right)\ \sin\theta d\theta-\frac{1}{2}\left(1+\frac{3}{ik_0 r}-\frac{3}{k_0^2 r^2}\right)\int_{shell}\sin^2\theta\exp\left(-ik_0 r\cos\theta\right)\ \sin\theta d\theta\right\} \quad \text{(S78)}$$

where $r = R_i + \delta_s / 2$. Using two integral identities

$$\int_0^{\pi}\exp\left(-ik_0 r\cos\theta\right)\sin\theta d\theta\ = 2\sin k_0 r / k_0 r \quad \text{(S79)}$$

and,

$$\int_S \exp(-ik_0 r\cos\theta)\ \sin^3\theta\ d\theta = \left(4\sin k_0 r - 4k_0 r\cos k_0 r\right)/ k_0^3 r^3 \quad \text{(S80)}$$

we can rewrite Equation S78 as:

$$\hat{y}\ \left(-i\kappa k_0^3\right)\frac{\exp(-ik_0 r)}{4\pi k_0 r}\left(2\pi r^2\delta_s\right)\left\{\left(1+\frac{1}{ik_0 r}-\frac{1}{k_0^2 r^2}\right)\left(\frac{2\sin k_0 r}{k_0 r}\right)-\frac{1}{2}\left(1+\frac{3}{ik_0 r}-\frac{3}{k_0^2 r^2}\right)\left(\frac{4\sin k_0 r-4k_0 r\cos k_0 r}{k_0^3 r^3}\right)\right\}$$

(S81)

The next term of interest is $-i\alpha_m^i\left(\kappa / c_0\right)\int_{shell}\overleftrightarrow{\boldsymbol{G}}_{EP}(0,\boldsymbol{r})\cdot\overleftrightarrow{\boldsymbol{G}}_{HM}(\boldsymbol{r},0)\cdot\boldsymbol{H}_{inc}(0)\, dv$. For this term, the incident magnetic field along y excites the magnetic dipole resonance of the NP in the same direction. That magnetic resonance produces a dipolar magnetic field at the position of the chiral shell. Then, the chirality of the shell (*i.e.*, the Pasteur parameter) converts the y-component of that dipolar magnetic field to electric dipole moments on the shell along y. Finally, it gives rise to the y-component of the local electric field at the position of the NP as:

$$\hat{y}\left(-i\alpha_m^i\kappa\right)\left(\frac{k_0^3\exp(-ik_0 r)}{4\pi k_0 r}\right)^2 \left\{\begin{aligned}&\left(1+\frac{3}{ik_0 r}-\frac{3}{k_0^2 r^2}\right)^2\left(r^2\delta_s\right)\int_{shell}\left(\sin\theta\cos\phi\right)^2\left(\sin\theta\sin\phi\right)^2\sin\theta d\theta d\phi+\left(1+\frac{1}{ik_0 r}-\frac{1}{k_0^2 r^2}\right)^2\left(4\pi r^2\delta_s\right)-\\ &2\left(1+\frac{1}{ik_0 r}-\frac{1}{k_0^2 r^2}\right)\left(1+\frac{3}{ik_0 r}-\frac{3}{k_0^2 r^2}\right)\left(r^2\delta_s\right)\int_{shell}\left(\sin\theta\sin\phi\right)^2\sin\theta d\theta d\phi\\ &+\left(1+\frac{3}{ik_0 r}-\frac{3}{k_0^2 r^2}\right)^2\left(r^2\delta_s\right)\int_{shell}\left(\sin\theta\sin\phi\right)^4\sin\theta d\theta d\phi+\\ &\left(1+\frac{3}{ik_0 r}-\frac{3}{k_0^2 r^2}\right)^2\left(r^2\delta_s\right)\int_{shell}\left(\sin\theta\sin\phi\right)^2\left(\cos\theta\right)^2\sin\theta d\theta d\phi\end{aligned}\right\} \quad \text{(S82)}$$

By calculating the integrals over the chiral shell, Equation S82 is simplified as:

$$\hat{y}\ \left(-i\,\alpha_m^i\kappa\right)\left(\frac{k_0^3\exp(-ik_0r)}{4\pi k_0 r}\right)^2\left(\frac{4\pi}{3}r^2\delta_s\right)\left(2+\frac{4}{ik_0r}-\frac{10}{\left(k_0r\right)^2}-\frac{12}{i\left(k_0r\right)^3}+\frac{6}{\left(k_0r\right)^4}\right) \qquad \text{(S83)}$$

The third term of interest is $i\left(\kappa/\eta_0\right)\int_{shell}\overleftrightarrow{\boldsymbol{G}}_{EM}(0,\boldsymbol{r})\cdot\boldsymbol{E}_{inc}(\boldsymbol{r})\,dv$, for which the physical origin of the non-zero local field along y is the magnetic dipole moments on the shell oriented along $x,$ that are induced by the x-polarized incident electric field. The y-component for this term is given by:

$$\hat{y}\ \left(i\,\kappa k_0^{\,3}\right)\frac{\exp(-ik_0r)}{4\pi k_0 r}\left(1+\frac{1}{ik_0r}\right)\left(2\pi r^2\delta_s\right)\int_{Shell}\exp\left(-ik_0r\cos\theta\right)\cos\theta\ \sin\theta d\theta \qquad \text{(S84)}$$

Using the integral identity $\int_0^\pi\exp(-ik_0r\cos\theta)\,\sin\theta\,\cos\theta d\theta\ =\frac{2i\left(k_0r\cos k_0r-\sin k_0r\right)}{k_0^2r^2}$, Equation S84 is simplified to:

$$\hat{y}\ \left(i\,\kappa k_0^{\,3}\right)\frac{\exp(-ik_0r)}{4\pi k_0 r}\left(1+\frac{1}{ik_0r}\right)\left(4\pi r^2\delta_s\right)\left(\frac{i\,\cos k_0r}{k_0r}-\frac{i\,\sin k_0r}{k_0^2r^2}\right) \qquad \text{(S85)}$$

Finally, the last term of interest is $i\,\alpha_m^i\left(\kappa/\eta_0\right)\int_{shell}\overleftrightarrow{\boldsymbol{G}}_{EM}(0,\boldsymbol{r})\cdot\overleftrightarrow{\boldsymbol{G}}_{EM}(\boldsymbol{r},0)\cdot\boldsymbol{H}_{inc}(0)\,dv$, for which the incident magnetic field excites the magnetic dipole of the NP along y. Then, the chirality of the shell converts the x-component of that dipolar electric field to magnetic dipole moments along x, which give rise to the y-component of the local electric field:

$$\hat{y}\ \left(i\,\alpha_m^i\kappa\right)\left(\frac{k_0^{\,3}\exp(-ik_0r)}{4\pi k_0 r}\right)^2\left(1+\frac{1}{ik_0r}\right)^2\left(4\pi r^2\delta_s\right)\left(\frac{2}{3}\right) \qquad \text{(S86)}$$

The total perturbative electric field along y (E_c in section S66) is given by the algebraic summation of Equations S81, S83, S85, and S86 which is related to the electric chirality transfer (*i.e.*, transfer of chirality through a change in local electric field). To obtain E_c , we first add Equations S81 and S84, which give the y-component of $-i\left(\kappa/c_0\right)\int_{shell}\overleftrightarrow{\boldsymbol{G}}_{EP}(0,\boldsymbol{r})\cdot\boldsymbol{H}_{inc}(\boldsymbol{r})\,dv+i\left(\kappa/\eta_0\right)\int_{shell}\overleftrightarrow{\boldsymbol{G}}_{EM}(0,\boldsymbol{r})\cdot\boldsymbol{E}_{inc}(\boldsymbol{r})\,dv$ as:

$$\hat{y}\ \left(-i\,\kappa k_0^3\right)\frac{\exp(-ik_0r)}{4\pi k_0 r}\left(4\pi r^2\delta_s\right)\left\{-i\,\frac{\exp\left(ik_0r\right)}{k_0r}-\frac{i}{k_0^{\,3}r^3}\left(3\cos k_0r-i\,\sin k_0r\right)-3\frac{\exp\left(-ik_0r\right)}{k_0^{\,4}r^4}+\frac{3\sin k_0r}{k_0^{\,5}r^5}\right\}$$

(S87)

and describes the electric chirality transfer caused by the incident field at the location of the chiral shell (*i.e.*, $\boldsymbol{E}_{inc}(\boldsymbol{r})$, $\boldsymbol{H}_{inc}(\boldsymbol{r})$). On the other hand, adding Equations S83 and S86 gives the y-component of

$-i\,\alpha_m\left(\kappa/c_0\right)\int_{shell}\overleftrightarrow{\boldsymbol{G}}_{EP}(0,\boldsymbol{r})\cdot\overleftrightarrow{\boldsymbol{G}}_{HM}(\boldsymbol{r},0)\cdot\boldsymbol{H}_{inc}(0)\,dv+i\,\alpha_m\left(\kappa/\eta_0\right)\int_{shell}\overleftrightarrow{\boldsymbol{G}}_{EM}(0,\boldsymbol{r})\cdot\overleftrightarrow{\boldsymbol{G}}_{EM}(\boldsymbol{r},0)\cdot\boldsymbol{H}_{inc}(0)\,dv$ as:

$$\hat{y}\ \left(i\,\alpha_m^i\kappa\right)\frac{k_0^{\,4}\delta_s}{6\pi}\exp(-2ik_0r)\left(\frac{4}{k_0^{\,2}r^2}-\frac{6i}{k_0^{\,3}r^3}-\frac{3}{k_0^{\,4}r^4}\right) \qquad \text{(S88)}$$

and accounts for the electric chirality transfer generated by the dipolar magnetic resonance. For high-index dielectric NPs, the electric chirality transfer is dominated by the dipolar term (Equation S88), and the incident term (Equation S87) can be neglected. Thus, the total E_c is almost equal to the value given in Equation S87.

II. Magnetic chirality transfer

For x-polarized illumination shown in Figure S7, the initial local magnetic field at the center of the NP is along y, but the chirality transfer effects (cross-polarization conversion) generate an x-component of small amplitude for the local magnetic field (H_c in Equation S66). To calculate this component, we write the magnetic counterpart of Equation S74 as:

$$\boldsymbol{H}(0)=\boldsymbol{H}_{inc}(0)+\int_{shell}\overleftrightarrow{\boldsymbol{G}}_{HP}(0,\boldsymbol{r})\cdot\left[\varepsilon_0\left(\varepsilon_r^c-1\right)\boldsymbol{E}\left(\boldsymbol{r}\right)-i\frac{\kappa}{c_0}\boldsymbol{H}\left(\boldsymbol{r}\right)\right]dv+\int_{shell}\overleftrightarrow{\boldsymbol{G}}_{HM}(0,\boldsymbol{r})\cdot\left[i\frac{\kappa}{\eta_0}\boldsymbol{E}\left(\boldsymbol{r}\right)\right]dv \quad \text{(S89)}$$

Then, by inserting $\boldsymbol{E}\left(\boldsymbol{r}\right)$ and $\boldsymbol{H}\left(\boldsymbol{r}\right)$ from Equations S75 and S76 (the electric and the magnetic fields at the position of the chiral shell), we obtain the local field as:

$$\begin{aligned}
&\boldsymbol{H}(0)=\boldsymbol{H}_{inc}(0)+\varepsilon_0\left(\varepsilon_r^c-1\right)\int_{shell}\overleftrightarrow{\boldsymbol{G}}_{HP}(0,\boldsymbol{r})\cdot\boldsymbol{E}_{inc}(\boldsymbol{r})\,dv+\alpha_e^i\,\varepsilon_0\left(\varepsilon_r^c-1\right)\int_{shell}\overleftrightarrow{\boldsymbol{G}}_{HP}(0,\boldsymbol{r})\cdot\overleftrightarrow{\boldsymbol{G}}_{EP}(\boldsymbol{r},0)\cdot\boldsymbol{E}_{inc}(0)\,dv+\\
&\alpha_m^i\,\varepsilon_0\left(\varepsilon_r^c-1\right)\int_{shell}\overleftrightarrow{\boldsymbol{G}}_{HP}(0,\boldsymbol{r})\cdot\overleftrightarrow{\boldsymbol{G}}_{EM}(\boldsymbol{r},0)\cdot\boldsymbol{H}_{inc}(0)\,dv-i\frac{\kappa}{c_0}\int_{shell}\overleftrightarrow{\boldsymbol{G}}_{HP}(0,\boldsymbol{r})\cdot\boldsymbol{H}_{inc}(\boldsymbol{r})\,dv-\\
&i\alpha_e^i\frac{\kappa}{c_0}\int_{shell}\overleftrightarrow{\boldsymbol{G}}_{HP}(0,\boldsymbol{r})\cdot\overleftrightarrow{\boldsymbol{G}}_{HP}(\boldsymbol{r},0)\cdot\boldsymbol{E}_{inc}(0)\,dv-i\alpha_m^i\frac{\kappa}{c_0}\int_{shell}\overleftrightarrow{\boldsymbol{G}}_{HP}(0,\boldsymbol{r})\cdot\overleftrightarrow{\boldsymbol{G}}_{HM}(\boldsymbol{r},0)\cdot\boldsymbol{H}_{inc}(0)\,dv+\\
&i\frac{\kappa}{\eta_0}\int_{shell}\overleftrightarrow{\boldsymbol{G}}_{HM}(0,\boldsymbol{r})\cdot\boldsymbol{E}_{inc}(\boldsymbol{r})\,dv+i\alpha_e^i\frac{\kappa}{\eta_0}\int_{shell}\overleftrightarrow{\boldsymbol{G}}_{HM}(0,\boldsymbol{r})\cdot\overleftrightarrow{\boldsymbol{G}}_{EP}(\boldsymbol{r},0)\cdot\boldsymbol{E}_{inc}(0)\,dv+\\
&i\alpha_m^i\frac{\kappa}{\eta_0}\int_{shell}\overleftrightarrow{\boldsymbol{G}}_{HM}(0,\boldsymbol{r})\cdot\overleftrightarrow{\boldsymbol{G}}_{EM}(\boldsymbol{r},0)\cdot\boldsymbol{H}_{inc}(0)\,dv
\end{aligned} \quad \text{(S90)}$$

Among the nine terms in Equation S90, only four generate a magnetic field component along x. These four terms are:

$$-i\left(\kappa/c_0\right)\int_{shell}\overleftrightarrow{\boldsymbol{G}}_{HP}(0,\boldsymbol{r})\cdot\boldsymbol{H}_{inc}(\boldsymbol{r})\,dv\;, \quad \text{(S91)}$$

$$-i\alpha_e^i\left(\kappa/c_0\right)\int_{shell}\overleftrightarrow{\boldsymbol{G}}_{HP}(0,\boldsymbol{r})\cdot\overleftrightarrow{\boldsymbol{G}}_{HP}(\boldsymbol{r},0)\cdot\boldsymbol{E}_{inc}(0)\,dv\;, \quad \text{(S92)}$$

$$i\left(\kappa/\eta_0\right)\int_{shell}\overleftrightarrow{\boldsymbol{G}}_{HM}(0,\boldsymbol{r})\cdot\boldsymbol{E}_{inc}(\boldsymbol{r})\,dv\;, \quad \text{(S93)}$$

and,

$$i\alpha_e^i\left(\kappa/\eta_0\right)\int_{shell}\overleftrightarrow{\boldsymbol{G}}_{HM}(0,\boldsymbol{r})\cdot\overleftrightarrow{\boldsymbol{G}}_{EP}(\boldsymbol{r},0)\cdot\boldsymbol{E}_{inc}(0)\,dv \quad \text{(S94)}$$

which produce the x-components of the local magnetic field:

$$\hat{x}\ \frac{-i\kappa k_0^{\,3}}{\eta_0}\frac{\exp(-ik_0r)}{4\pi k_0 r}\left(1+\frac{1}{ik_0r}\right)\left(4\pi r^2\delta_s\right)\left(\frac{i\cos k_0r}{k_0r}-\frac{i\sin k_0r}{k_0^2r^2}\right)\qquad\text{(S95)}$$

$$\hat{x}\ \left(-i\alpha_e^i\kappa c_0\right)\left(\frac{k_0^{\,3}\exp(-ik_0r)}{4\pi k_0 r}\right)^2\left(1+\frac{1}{ik_0r}\right)^2\left(4\pi r^2\delta_s\right)\left(\frac{2}{3}\right)\qquad\text{(S96)}$$

$$\hat{x}\ \frac{i\kappa k_0^3}{\eta_0}\frac{\exp(-ik_0r)}{4\pi k_0 r}\left(4\pi r^2\delta_s\right)\left\{\left(1+\frac{1}{ik_0r}-\frac{1}{k_0^2r^2}\right)\left(\frac{\sin k_0r}{k_0r}\right)-\left(1+\frac{3}{ik_0r}-\frac{3}{k_0^2r^2}\right)\left(\frac{\sin k_0r}{k_0^3r^3}-\frac{\cos k_0r}{k_0^2r^2}\right)\right\}\text{(S97)}$$

$$\hat{x}\ \left(i\alpha_e^i\kappa c_0\right)\left(\frac{k_0^3\exp(-ik_0r)}{4\pi k_0 r}\right)^2\left(\frac{4\pi}{3}r^2\delta_s\right)\left(2+\frac{4}{ik_0r}-\frac{10}{\left(k_0r\right)^2}-\frac{12}{i\left(k_0r\right)^3}+\frac{6}{\left(k_0r\right)^4}\right)\qquad\text{(S98)}$$

respectively. The total perturbative magnetic field in the *x*-direction is a direct summation of Equations S95-S98, which is related to the magnetic chirality transfer (transfer of chirality through the change in the local magnetic field). To find H_c, we first add Equations S95 and S97, which gives:

$$\hat{x}\ \frac{i\kappa k_0^3}{\eta_0}\frac{\exp(-ik_0r)}{4\pi k_0 r}\left(4\pi r^2\delta_s\right)\left\{-i\,\frac{\exp\left(ik_0r\right)}{k_0r}-\frac{i}{k_0^{\,3}r^3}\left(3\cos k_0r-i\sin k_0r\right)-3\frac{\exp\left(-ik_0r\right)}{k_0^{\,4}r^4}+\frac{3\sin k_0r}{k_0^{\,5}r^5}\right\}\text{(S99)}$$

Equation S99 describes the incident part of the magnetic chirality transfer (caused by the incident field at the location of the chiral shell). On the other hand, adding Equations S96 and S98 gives:

$$\hat{x}\ \left(-i\alpha_e^i\kappa c_0\right)\left(\frac{k_0^4\delta_s}{6\pi}\right)\exp(-2ik_0r)\left(\frac{4}{k_0^{\,2}r^2}-\frac{6i}{k_0^{\,3}r^3}-\frac{3}{k_0^{\,4}r^4}\right)\qquad\text{(S100)}$$

which accounts for the dipolar part of the magnetic chirality transfer. For high-index dielectric NPs, the dipolar part (Equation S100) dominates over the incident part (Equation S99), and the total H_c is almost equal to the value given in Equation S100.

III. Total chirality transfer

Rewriting the perturbative electric and the magnetic fields in terms of the Mie coefficients gives E_c and H_c as:

$$E_c\simeq\hat{y}\ \left(k_0\ \delta_s\ \kappa\ b_1\right)\exp(-2ik_0r)\left(\frac{4}{k_0^{\,2}r^2}-\frac{6i}{k_0^{\,3}r^3}-\frac{3}{k_0^{\,4}r^4}\right)\qquad\text{(S101)}$$

$$H_c\simeq\hat{x}\ \left(-\frac{k_0\delta_s\kappa}{\eta_0}a_1\right)\exp(-2ik_0r)\left(\frac{4}{k_0^{\,2}r^2}-\frac{6i}{k_0^{\,3}r^3}-\frac{3}{k_0^{\,4}r^4}\right)\qquad\text{(S102)}$$

where we have assumed that the dipolar part of either electric or magnetic chirality transfer dominates over the incident part. Now, if we assume that the system in Figure S7 is illuminated sequentially with right- and

left-handed circularly polarized plane waves, the differential intensity of the local electric ($\Delta|\boldsymbol{E}(0)|^2$) and local magnetic ($\Delta|\boldsymbol{H}(0)|^2$) fields are expressed as (Equations S69 and S70):

$$\Delta|\boldsymbol{E}(0)|^2 = -E_0\left(8k_0\delta_s\right)\mathrm{Im}\left(\kappa\ b_1\ \exp(-2ik_0r)\left(\frac{4}{k_0^{\ 2}r^2}-\frac{6i}{k_0^{\ 3}r^3}-\frac{3}{k_0^{\ 4}r^4}\right)\right) \quad \text{(S103)}$$

$$\Delta|\boldsymbol{H}(0)|^2 = H_0\left(\frac{8k_0\delta_s}{\eta_0}\right)\mathrm{Im}\left(\kappa\ a_1\ \exp(-2ik_0r)\left(\frac{4}{k_0^{\ 2}r^2}-\frac{6i}{k_0^{\ 3}r^3}-\frac{3}{k_0^{\ 4}r^4}\right)\right) \quad \text{(S104)}$$

Next, by putting these equations in Equations S45 and S46, we can calculate the differential extinct and the differential scattered powers of the NP as:

$$\Delta P_{ext}^{i} = \frac{24\pi E_0}{\eta_0 k_0^2}\left(k_0\delta_s\right)\left\{\begin{array}{l}\mathrm{Re}\left(a_1\right)\mathrm{Im}\left[\kappa\ b_1\left(\frac{4}{k_0^{\ 2}r^2}-\frac{6i}{k_0^{\ 3}r^3}-\frac{3}{k_0^{\ 4}r^4}\right)\exp(-2ik_0r)\right]+\\ \mathrm{Re}\left(b_1\right)\mathrm{Im}\left[\kappa\ a_1\left(\frac{4}{k_0^{\ 2}r^2}-\frac{6i}{k_0^{\ 3}r^3}-\frac{3}{k_0^{\ 4}r^4}\right)\exp(-2ik_0r)\right]\end{array}\right\} \quad \text{(S105)}$$

and,

$$\Delta P_{sca}^{i} = \frac{24\pi E_0}{\eta_0 k_0^2}\left(k_0\delta_s\right)\left\{\begin{array}{l}|a_1|^2\ \mathrm{Im}\left[\kappa\ b_1\left(\frac{4}{k_0^{\ 2}r^2}-\frac{6i}{k_0^{\ 3}r^3}-\frac{3}{k_0^{\ 4}r^4}\right)\exp(-2ik_0r)\right]+\\ |b_1|^2\ \mathrm{Im}\left[\kappa\ a_1\left(\frac{4}{k_0^{\ 2}r^2}-\frac{6i}{k_0^{\ 3}r^3}-\frac{3}{k_0^{\ 4}r^4}\right)\exp(-2ik_0r)\right]\end{array}\right\} \quad \text{(S106)}$$

respectively. To further simplify the results, we use the Taylor series of the r-dependent term in Equations S105 and S106:

$$\left(\frac{4}{k_0^{\ 2}r^2}-\frac{6i}{k_0^{\ 3}r^3}-\frac{3}{k_0^{\ 4}r^4}\right)\exp(-2ik_0r) \simeq \left(-\frac{3}{k_0^{\ 4}r^4}-\frac{2}{k_0^{\ 2}r^2}-2+\frac{32i}{15}k_0r+\frac{4}{3}k_0^{\ 2}r^2-\frac{64i}{105}k_0^{\ 3}r^3\right) \quad \text{(S107)}$$

The lowest order imaginary term is five orders of magnitude weaker than the lowest order real term, and thus for spheres with $k_0r<1$, it is reasonable to approximate the radius dependence as being purely real:

$$\left(\frac{4}{k_0^{\ 2}r^2}-\frac{6i}{k_0^{\ 3}r^3}-\frac{3}{k_0^{\ 4}r^4}\right)\exp(-2ik_0r) \simeq -\left(\frac{3}{k_0^{\ 4}r^4}+\frac{2}{k_0^{\ 2}r^2}+2\right) \quad \text{(S108)}$$

This renders the normalized differential extinct and scattered powers in Equations S105 and S106 as:

$$\frac{\Delta P_{ext}^{i}}{\Delta P_0^{s}} = \frac{-1}{\mathrm{Im}(\kappa)}\left(\frac{3}{k_0^2r^2}+\frac{3}{k_0^{\ 4}r^4}+\frac{9}{2k_0^{\ 6}r^6}\right)\left[\mathrm{Re}\left(a_1\right)\mathrm{Im}\left(\kappa b_1\right)+\mathrm{Re}\left(b_1\right)\mathrm{Im}\left(\kappa a_1\right)\right] \quad \text{(S109)}$$

$$\frac{\Delta P_{sca}^{i}}{\Delta P_0^{s}} = \frac{-1}{\mathrm{Im}(\kappa)}\left(\frac{3}{k_0^2r^2}+\frac{3}{k_0^{\ 4}r^4}+\frac{9}{2k_0^{\ 6}r^6}\right)\mathrm{Im}\left[\kappa\ a_1b_1\left(a_1^*+b_1^*\right)\right] \quad \text{(S110)}$$

where $\Delta P_0^s = 4k_0 \operatorname{Im}(\kappa) V_{shell} / \eta_0$ is the differential extinct power of the chiral shell in free space and $V_{shell} = 4\pi r^2 \delta_s$ is the volume of the thin chiral shell.

To investigate the validity of these equations, we consider a dielectric nanosphere of radius 50 nm covered by a thin chiral shell of thickness 10 nm. We calculate the differential absorption, scattering, and extinction of the nanosphere using full-wave numerical simulation (see Supporting Section S8) and compare the results to the values obtained from closed-form derivations in Equations S109 and S110 (Figure S8). To keep the numerical error above a certain level and to avoid noisy data, we increase the calibrated permittivity and the Pasteur parameter in Equations S8 and S9 by the factor of 10^4. The good agreement proves the validity of the approximations in deriving Equations S109 and S110. Finally, we compare the results to a plasmonic particle of the same radius (yellow curve in Figure S8). We see that the chirality transfer to the dielectric system is significantly higher than that of the plasmonic one.

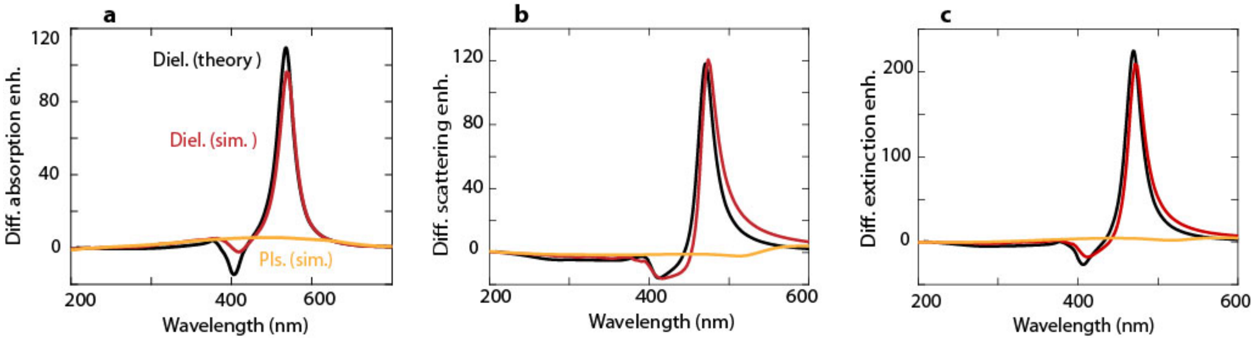

Figure S8. Differential absorption (a), differential scattering (b), and differential extinction (c) of dielectric and plasmonic spheres of radius 50 nm covered by a thin chiral shell of thickness 10 nm. The dielectric and the plasmonic spheres are made of silicon and gold. The Pasteur parameter and the permittivity of the chiral shell are taken from the calibrated Lorentzian model Equations S8 and S9, but for these results β and γ are magnified by a factor 10^4. In each panel, red and black represent simulation and theory (Equations S109 and S110) for dielectric nanosphere, respectively, while yellow shows a calculation using a full-wave numerical simulation for plasmonic nanoparticle.

Supporting Section S5. Fundamental limits of chirality transfer for a small chiral sphere near an achiral nanoparticle

Equation S63 describes the differential extinction of an achiral nanoparticle close to a small chiral sphere in terms of the field enhancement at the position of the chiral sphere. We can express that near field in terms of the incident plus the dipolar fields due to the NP as:

$$E_{y,R}^{c,enh} = 1 - i\,6\pi\zeta\left(k_0 l\right) a_1 \tag{S111}$$

$$H_{y,R}^{c,enh} = 1 - i\,6\pi\zeta\left(k_0 l\right) b_1 \tag{S112}$$

Substituting Equations S111 and S112 into Equation S63 results in the differential extinction as:

$$\begin{aligned}\frac{\Delta P_{ext}^i}{\Delta P_0} = &\frac{-3\operatorname{Re}(\kappa)}{2\operatorname{Im}(\kappa)}\left(\frac{1}{k_0^3 l^3}+\frac{1}{2k_0 l}\right)\left[\operatorname{Re}(a_1)+\operatorname{Re}(b_1)\right]- \\ &\frac{9\pi\operatorname{Re}(\kappa)}{\operatorname{Im}(\kappa)}\left(\frac{1}{k_0^3 l^3}+\frac{1}{2k_0 l}\right)\left[\operatorname{Re}(a_1)\operatorname{Im}\left(\zeta\left(k_0 l\right)b_1\right)+\operatorname{Re}(b_1)\operatorname{Im}\left(\zeta\left(k_0 l\right)a_1\right)\right]\end{aligned} \tag{S113}$$

Using the Taylor expansion of $\zeta(k_0 l) \simeq \frac{1}{2\pi}\left(\frac{1}{k_0^3 l^3}+\frac{1}{2k_0 l}\right)$, we can write Equation S113 as:

$$\begin{aligned}\frac{\Delta P_{ext}^{i}}{\Delta P_0} &= \frac{-3\,\mathrm{Re}(\kappa)}{2\,\mathrm{Im}(\kappa)}\left(\frac{1}{k_0^3 l^3}+\frac{1}{2k_0 l}\right)\left[\mathrm{Re}(a_1)+\mathrm{Re}(b_1)\right] \\ &-\frac{9\,\mathrm{Re}(\kappa)}{2\,\mathrm{Im}(\kappa)}\left(\frac{1}{k_0^6 l^6}+\frac{1}{k_0^4 l^4}+\frac{1}{4k_0^2 l^2}\right)\left[\mathrm{Re}(a_1)\mathrm{Im}(b_1)+\mathrm{Re}(b_1)\mathrm{Im}(a_1)\right]\end{aligned} \tag{S114}$$

The first and the second terms in Equation S114 come from the incident and the dipolar fields, respectively. Far from the intrinsic molecular resonance, the imaginary part of the Pasteur parameter is negligible compared to its real part. On the other side of the equation, the maximum value for $\mathrm{Re}(a_1)+\mathrm{Re}(b_1)$ and $\mathrm{Re}(a_1)\mathrm{Im}(b_1)+\mathrm{Re}(b_1)\mathrm{Im}(a_1)$ is 2 and $3\sqrt{3}/8$, which occurs when conditions $a_1 = b_1 = 1$ and $\mathrm{Re}(a_1)=\mathrm{Re}(b_1)=3/4$ are satisfied, respectively (see Supporting Section S6). This provides an upper limit of the incident and the dipolar terms:

$$\left(\frac{\Delta P_{ext}^{i}}{\Delta P_0}\right)_{inc} \le -3\frac{\mathrm{Re}(\kappa)}{\mathrm{Im}(\kappa)}\left(\frac{1}{k_0^3 l^3}+\frac{1}{2k_0 l}\right) \tag{S115}$$

and,

$$\left(\frac{\Delta P_{ext}^{i}}{\Delta P_0}\right)_{dip} \le -\frac{27\sqrt{3}}{16}\frac{\mathrm{Re}(\kappa)}{\mathrm{Im}(\kappa)}\left(\frac{1}{k_0^6 l^6}+\frac{1}{k_0^4 l^4}+\frac{1}{4k_0^2 l^2}\right) \tag{S116}$$

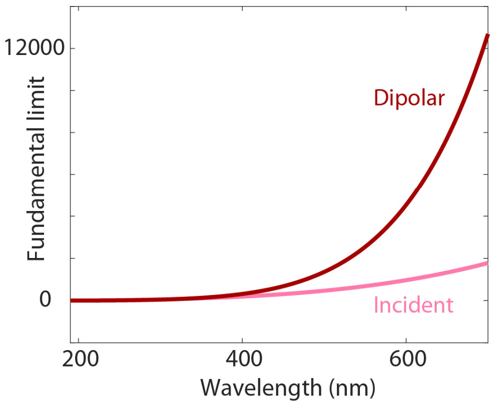

Figure S9. The incident (Equation S115) and the dipolar (Equation S116) fundamental limits of chirality transfer for a chiral sphere near an achiral NP. The radius of the NP and chiral sphere are 50 nm and 5 nm, respectively, and the gap distance between them is 5 nm ($l = 60$ nm). The permittivity and the Pasteur parameter are taken from the calibrated values in Equations S8 and S9.

respectively. In Figure S9, we plot the incident and the dipolar limits for a chiral sphere of radius 5 nm placed at a center-to-center distance of 60 nm to an achiral NP of radius 50 nm. We see that the dipolar limit is much higher than the incident one.

Supporting Section S6. Fundamental limits of chirality transfer for an achiral NP covered by a thin chiral shell

To find the fundamental limits of differential extinction, we consider two cases based on the relative magnitude of the real to the imaginary part of the Pasteur parameter. The first one is close to the intrinsic molecular resonance where $\mathrm{Re}(\kappa) \ll \mathrm{Im}(\kappa)$, for which we can rewrite Equations S109 as:

$$\frac{\Delta P_{ext}^{i}}{\Delta P_{0}^{s}} = -2\left(\frac{3}{k_0^2 r^2} + \frac{3}{k_0^4 r^4} + \frac{9}{2k_0^6 r^6}\right)\mathrm{Re}(a_1)\mathrm{Re}(b_1) \qquad \text{(S117)}$$

From the optical theorem, we know that $\mathrm{Re}(a_1, b_1) \geq |a_1, b_1|^2$. We also know that the upper limit of the Mie coefficients is 1.[10] These two conditions together provide the upper limit of differential extinction:

$$\frac{\Delta P_{ext}^{i}}{\Delta P_{0}^{s}} \leq -2\left(\frac{3}{k_0^2 r^2} + \frac{3}{k_0^4 r^4} + \frac{9}{2k_0^6 r^6}\right) \qquad \text{(S118)}$$

On the other hand, far from the molecular resonance $\mathrm{Re}(\kappa) \geq \mathrm{Im}(\kappa)$ and, thus, Equation S109 can be expressed as:

$$\frac{\Delta P_{ext}^{i}}{\Delta P_{0}^{s}} = -\frac{\mathrm{Re}(\kappa)}{\mathrm{Im}(\kappa)}\left(\frac{3}{k_0^2 r^2} + \frac{3}{k_0^4 r^4} + \frac{9}{2k_0^6 r^6}\right)\left[\mathrm{Re}(a_1)\mathrm{Im}(b_1) + \mathrm{Re}(b_1)\mathrm{Im}(a_1)\right] \qquad \text{(S119)}$$

which can be rewritten as:

$$\frac{\Delta P_{ext}^{i}}{\Delta P_{0}^{s}} = -\frac{\mathrm{Re}(\kappa)}{\mathrm{Im}(\kappa)}\left(\frac{3}{k_0^2 r^2} + \frac{3}{k_0^4 r^4} + \frac{9}{2k_0^6 r^6}\right)\left[\mathrm{Re}(a_1)\sqrt{|b_1|^2 - \left(\mathrm{Re}(b_1)\right)^2} + \mathrm{Re}(b_1)\sqrt{|a_1|^2 - \left(\mathrm{Re}(a_1)\right)^2}\right]$$

(S120)

Here, again, from the optical theorem we have $\mathrm{Re}(a_1, b_1) \geq |a_1, b_1|^2$ and we can write the following inequality:

$$\frac{\Delta P_{ext}^{i}}{\Delta P_{0}^{s}} \leq -\frac{\mathrm{Re}(\kappa)}{\mathrm{Im}(\kappa)}\left(\frac{3}{k_0^2 r^2} + \frac{3}{k_0^4 r^4} + \frac{9}{2k_0^6 r^6}\right)\left[\mathrm{Re}(a_1)\sqrt{\mathrm{Re}(b_1) - \left(\mathrm{Re}(b_1)\right)^2} + \mathrm{Re}(b_1)\sqrt{\mathrm{Re}(a_1) - \left(\mathrm{Re}(a_1)\right)^2}\right]$$

(S121)

It can be shown that the extremum of the Mie-coefficient-dependent term is $3\sqrt{3}/8$, which yields the following fundamental limit:

$$\frac{\Delta P_{ext}^{i}}{\Delta P_{0}^{s}} \leq -\frac{3\sqrt{3}}{8}\frac{\mathrm{Re}(\kappa)}{\mathrm{Im}(\kappa)}\left(\frac{3}{k_0^2 r^2} + \frac{3}{k_0^4 r^4} + \frac{9}{2k_0^6 r^6}\right) \qquad \text{(S122)}$$

Supporting Section S7. Beer-Lambert law and calibration of the Lorentzian model

To calibrate the parameters in the Lorentzian model in Equations S8 and S9 for realistic simulations, we use typical values of the differential and the mean molar extinction cross-section for proteins, which are

$\Delta\varepsilon = 20$ M^{-1}cm^{-1} and $\bar{\varepsilon} = 10^4$ M^{-1}cm^{-1}, respectively (dissymmetry factor of $g = \Delta\varepsilon/\bar{\varepsilon} = 0.002$).[5,11] To see how β and γ can be related to $\Delta\varepsilon$ and $\bar{\varepsilon}$, we consider a chiral slab of thickness l illuminated by right - and left-handed circularly polarized light (RCP and LCP, respectively) as $\boldsymbol{E}_{R/L}\left(z=0\right) = E_0 \exp(-ik_0 z)\,\hat{e}_{R/L}$, where $\hat{e}_R = \left(\hat{x} - i\hat{y}\right)$ and $\hat{e}_L = \left(\hat{x} + i\hat{y}\right)$ are the base vectors for the right- and left-handed circular polarizations, respectively.

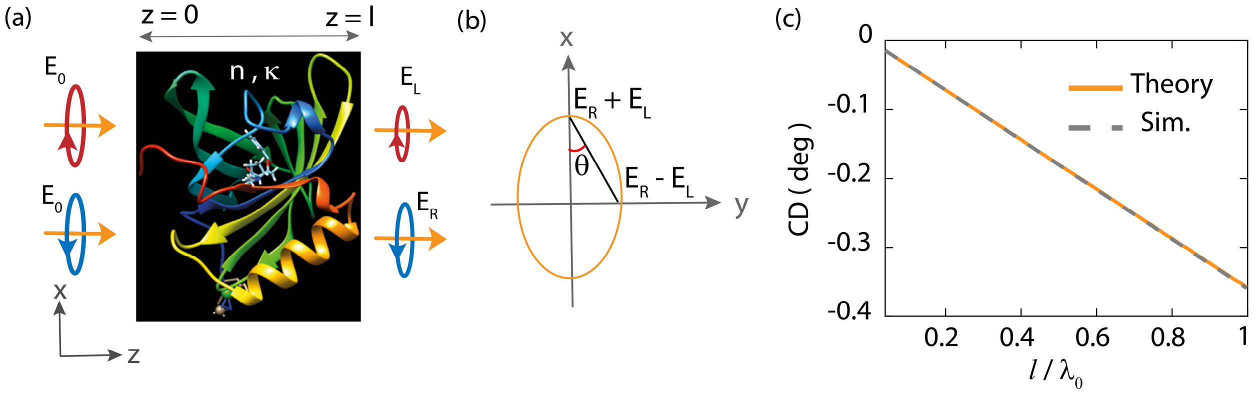

Figure S10. (a) A chiral slab of thickness l illuminated by right- and left-handed circularly polarized light of equal amplitudes E_0 . E_R and E_L denote the amplitudes of the corresponding electric fields at the output side of the slab. (b) Interpretation of the CD as ellipticity angle θ . (c) Analytical (Equation S125) and simulated CD as a function of thickness for a chiral slab of refractive index $n = 1.45 - 0.01i$ and Pasteur parameter $\kappa = 0.01 - 0.001i$ at $\lambda_0 = 500$ nm .

The chiral slab has a refractive index n and Pasteur parameter κ . Thus, the effective refractive indices for RCP and the LCP lights propagating through the slab are $n_R = n + \kappa$ and $n_L = n - \kappa$, respectively. This renders the corresponding fields at the output side:

$$\boldsymbol{E}_{R/L}\left(z = l\right) = E_0 \exp(-ik_0 n_{R/L} l)\,\hat{e}_{R/L} \qquad \text{(S123)}$$

Generally, when both the real and imaginary parts of the Pasteur parameter are considered, the superposition of the electric fields at the output plane creates a rotated ellipse. However, close to the intrinsic resonance, the imaginary part is dominant, and thus the rotation is negligible. In this case, if E_R and E_L are the magnitudes of the electric field vectors for RCP and LCP output light, then the CD is reported in units of degrees as the ellipticity θ (see Figure S10b) expressed as:

$$CD = \tan^{-1}\left(\frac{E_R - E_L}{E_R + E_L}\right) \qquad \text{(S124)}$$

where E_R and E_L are the amplitudes of the right- and left-handed circularly polarized light at the output side as $E_{R/L} = \left|\boldsymbol{E}_{R/L}\left(z = l\right)\right|$. The CD in Equation S124 takes values of 0 and $\pm\pi/4$ for non-chiral and perfectly chiral media, respectively. By putting the electric field amplitudes from Equation S123 in Equation S124 and assuming small ellipticity ($\tan\theta \simeq \theta$), we obtain the following expression for the CD signal:

$$CD\left(\deg\right) \simeq k_0 \mathrm{Im}\left(\kappa\right)\ l \left(\frac{180}{\pi}\right) \qquad \text{(S125)}$$

Figure S10c shows CD as a function of the thickness for a chiral slab of the Pasteur parameter $\kappa = 0.01 - 0.001i$ and refractive index $n = 1.45 - 0.01i$ at a wavelength $\lambda_0 = 500\ \mathrm{nm}$. We see that the value obtained from Equation S125 (grey curve) is in perfect agreement with the simulation (orange).

According to the Beer-Lambert law, the optical attenuation of light for a chiral sample of uniform concentration can be expressed as:

$$A_{R/L} = \varepsilon_{R/L}\ C\ l \qquad \text{(S126)}$$

where "*R*" and "*L*" subscripts refer to the two circular polarizations of illumination and A is the absorbance of the sample, which is defined as the decadic logarithm of the ratio of the incident (I_0) to the transmitted power intensity (I) as $A = \log\left(I_0/I\right)$. C is the molar concentration in mol/L (usually denoted by *M*) and l is the path length in cm. ε is the molar extinction cross-section in $\mathrm{M^{-1}cm^{-1}}$ which is related to the extinction cross-section of species inside the sample (σ in $\mathrm{cm^2}$) as $\varepsilon = \sigma\left(2.6157\times 10^{20}\right)$. Noting that the absorbances for RCP and LCP (*i.e.*, A_R and A_L) are connected to the amplitudes of E_R and E_L (Equation S123) as $E_{R/L} = \sqrt{I_0}\ \exp\left(-A_{R/L}\ \ln\left(10\right)/2\right)$, the CD (in degrees) can be rewritten in terms of the differential absorbance ($\Delta A = A_R - A_L$) as:

$$CD = \Delta A\ \frac{\ln\left(10\right)}{4}\ \frac{180}{\pi} = 32.98\ \Delta A \qquad \text{(S127)}$$

Next, combining Equation S126 and Equation S127 gives:

$$CD = 32.98\ \Delta\varepsilon\ C\ l \qquad \text{(S128)}$$

where $\Delta\varepsilon = \Delta\sigma\left(2.6157\times 10^{20}\right)$ is the differential molar attenuation of the species inside the chiral sample in $\mathrm{cm^2/mol}$, with $\Delta\sigma$ the differential extinction cross-section of the species inside the chiral solution in $\mathrm{cm^2}$. Finally, by equating Equations S125 and S128, we can rewrite the imaginary part of the Pasteur parameter as:

$$\mathrm{Im}\left(\kappa\right) = \left(\frac{32.98}{360}\right)\ \lambda_0\ \Delta\varepsilon\ C \qquad \text{(S129)}$$

where λ_0 is the wavelength in cm. If V_c is the volume of chiral sample in each unit-cell (in units of liters) and *n* is the number of molecules per unit cell, then *C* can be determined from:

$$C = \frac{n_c / N_A}{V_c} \qquad \text{(S130)}$$

Substituting Equation S130 in Equation S129 gives:

$$\mathrm{Im}\left(\kappa\right) = 0.0916\ \frac{\lambda_0\ \Delta\varepsilon\ n_c}{N_A\ V_c} \qquad \text{(S131)}$$

Now, we consider a typical value of $\Delta\varepsilon = 20$ M^{-1}cm^{-1} at $\lambda_0 = 220$ nm .[12] Table S1 shows the calculated values of $\mathrm{Im}(\kappa)$ and the corresponding molar concentrations for different values of n_c. For this calculation, we considered our proposed metasurface (see Figure 4 in the main manuscript), in which the lattice constant is 320 nm, and the thickness of the chiral layer is 10 nm. The final calibration step is to tune the chirality parameter β in Equation S9 for a given imaginary part of the Pasteur parameter, which leads to β=6.53×10^{-9} eV for $n_c = 1000$.

Table S1. Molar concentration and imaginary part of the Pasteur parameter for different numbers of chiral molecules in each unit cell.

n_c (number of chiral molecules in each unit cell)	C (M) molar concentration	$\mathrm{Im}(\kappa)$
1	1.6 µM	6.5×10^{-11}
10	16 µM	6.5×10^{-10}
100	160 µM	6.5×10^{-9}
1000	1.6 mM	6.5×10^{-8}

In the same way that the ellipticity was connected to the imaginary part of $\mathrm{Im}(\kappa)$, we can link the normalized mean electric field to the imaginary part of the refractive index *n* as:

$$\frac{E_R + E_L}{2E_0} = \exp\left(k_0 \,\mathrm{Im}(n)\, l\right) \qquad \text{(S132)}$$

in which we have assumed that $\left|\mathrm{Im}(n)\right| \gg \left|\mathrm{Im}(\kappa)\right|$.

Then, recalling that $E_{R/L} = \sqrt{I_0}\,\exp\left(-A_{R/L}\,\ln(10)/2\right)$, the same quantity in Equation S132 is expressed in terms of the mean absorbance $\bar{A} = \left(A_R + A_L\right)/2$ as:

$$\frac{E_R + E_L}{2E_0} = 1 - \bar{A}\,\frac{\ln(10)}{2} \qquad \text{(S133)}$$

Equating Equations S132 and S133 and then using the Beer-Lambert law (Equation S126), gives the mean molar extinction cross-section $\bar{\varepsilon} = \left(\varepsilon_R + \varepsilon_L\right)/2$:

$$\bar{\varepsilon} = \frac{1 - \exp\left(k_0 \,\mathrm{Im}(n)\, l\right)}{\frac{\ln(10)}{2} C\; l} \qquad \text{(S134)}$$

Assuming $C = 1.6$ mM ($n_c = 1000$) and a typical value of $\bar{\varepsilon} = 10^4$ M^{-1}cm^{-1}, gives us a calibrated value for the absorption parameter in the Lorentzian model γ=1.8×10^{-5} eV. Figures S11a, b show the calibrated permittivity and the Pasteur parameter, respectively, obtained from the Lorentzian model in Equations S8

and S9. Figures S11c, d show the differential and the mean molar extinction cross sections using the calibrated values in Figures S11a, b, taking the values of $\Delta\varepsilon = 20$ M^{-1}cm^{-1} and $\bar{\varepsilon} = 2\times10^4$ M^{-1}cm^{-1} at a resonance wavelength of 220 nm. In Figures S11e, f, the CD and the differential absorbance are plotted for a chiral slab of path length $l = 10$ nm with the Pasteur parameter and refractive index given in Figures S11a,b.

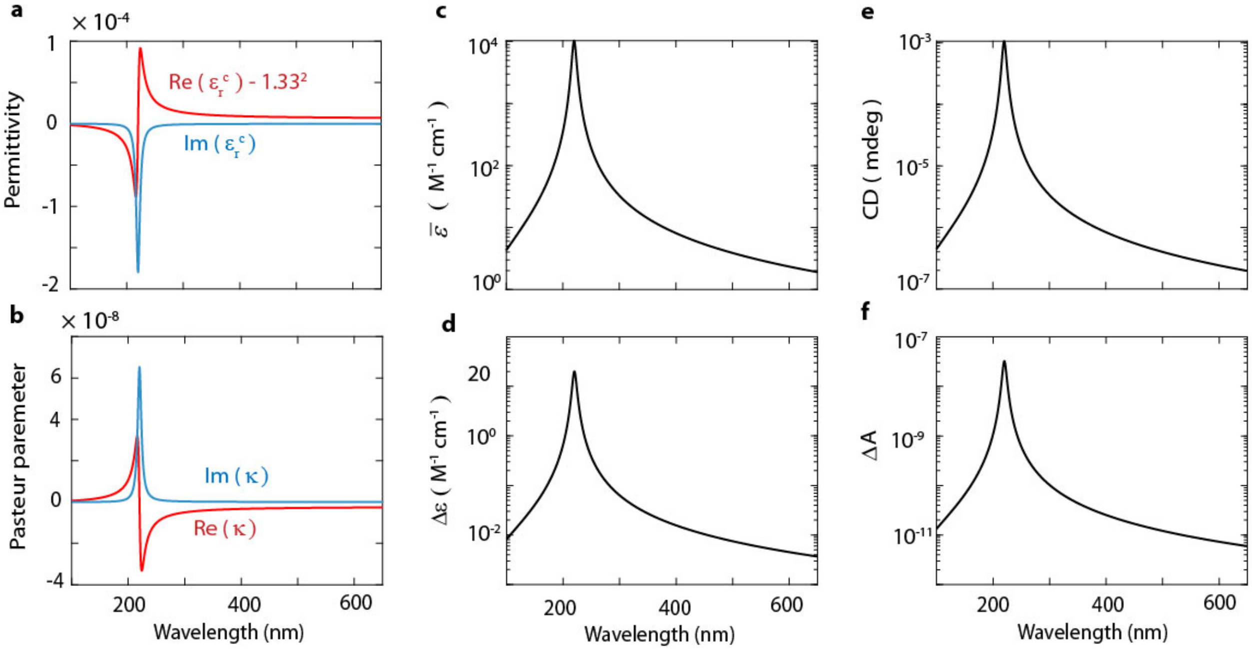

Figure S11. Calibration of the Lorentzian model in Equations S8 and S9 for the typical values of $\Delta\varepsilon = 20$ M^{-1}cm^{-1} and $\bar{\varepsilon} = 10^4$ M^{-1}cm^{-1} at $\lambda_0 = 220$ nm, which gives γ=1.8×10^{-5} eV, β=6.53×10^{-9} eV, and Γ= 0.1 eV. The molar concentration is $C = 1.6$ mM (Equation S130 with $n_c = 1000$). Real (red) and imaginary (blue) parts of the calibrated (a) permittivity and (b) the Pasteur parameter. (c) The mean (Equation S134) and (d) the differential (Equation S131) molar extinction cross sections. (e) The CD and (f) the differential absorbance of a chiral sample of path length $l = 10$ nm (Equation S127).

Supporting Section S8. Numerical simulations

For numerical simulations, we used the frequency-domain solver of COMSOL Multiphysics 5.5, which exploits the finite element method (FEM). We modified the default built-in wave equations to model the constitutive equations in Equation S71-S73.[13] The silicon and gold permittivities are taken from experimental data.[7,8] The refractive index of the substrate and the buffer solution are set to 1.5 and 1.33, respectively. The mesh size for the chiral film and the silicon disk is set to 5 and 20 nm, respectively. To suppress numerical artifacts in the combined system of nanostructure and chiral film, we used a mirror-symmetric mesh for both the chiral film and the nanodisks.[14]

Supporting Section S9. Circular dichroism definition

For the combined system of chiral film and metasurface in Figure 4 of the main manuscript, the CD can be reported in terms of the ellipticity (see Figure S11b) as:

$$CD = \tan^{-1}\left(\frac{\sqrt{T_+} - \sqrt{T_-}}{\sqrt{T_+} + \sqrt{T_-}}\right) \qquad \text{(S135)}$$

where T_+ and T_+ are the transmission coefficients for illumination under the right- and left-handed circular polarization. Usually, the difference between these transmission coefficients is very small ($T_+ \simeq T_-$), which provides Equation S135 as:

$$CD \simeq \frac{T_+ - T_-}{4T} \qquad \text{(S136)}$$

where T in the denominator can be either T_+ or T_-. On the other hand, the differential transmittance of the system ($\Delta T = T_+ - T_-$) is related to the differential absorptance ($\Delta A = A_+ - A_-$) and the differential reflectance ($\Delta R = R_+ - R_-$) as:

$$\Delta T = -\Delta R - \Delta A \qquad \text{(S137)}$$

Figure S12 shows ΔT (red) and ΔR (blue) for the combined system of the metasurface and chiral film in Figure 4 of the main manuscript. We see that the differential reflectance is negligible compared to the differential transmittance. This simplifies Equation S137 as:

$$\Delta T \simeq -\Delta A \qquad \text{(S138)}$$

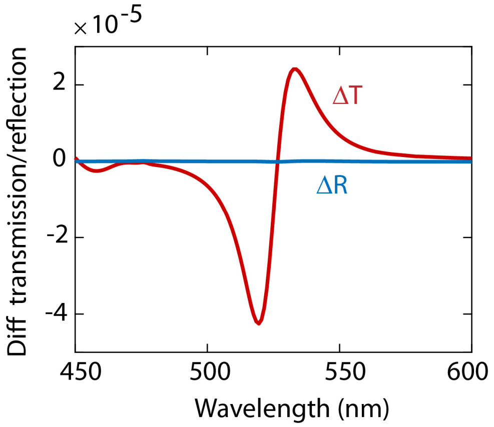

Figure S12. The differential transmission ($\Delta T = T_+ - T_-$) and the differential reflection ($\Delta R = R_+ - R_-$) of the metasurface in Figure 4 of the main manuscript with $h = 70nm$, $R = 60nm$, $w_i = 10nm$, $P = 320\ nm$. The Pasteur parameter and the permittivity of the chiral layer are taken from the calibrated Lorentzian model in section S7 but β and γ are magnified by a factor of 10^4 for numerical robustness.

Next, the differential absorptance is related to the total differential absorbed power in a unit cell of the metasurface by:[15]

$$\Delta A = \frac{-1}{P_{inc}}\left(\Delta P_{abs}^{i} + \Delta P_{abs}^{c}\right) \qquad \text{(S139)}$$

where ΔP_{abs}^{i} and ΔP_{abs}^{c} are the differential absorbed powers inside one single disk (*i.e.*, chirality transfer) and inside the chiral film placed on top of that disk, respectively. $P_{inc} = E_0^2 A / \eta_0$ is the incident power of the circularly polarized illumination passing through the area of the unit cell (*A)*. Replacing ΔT in Equation S136 by $-\Delta A$ from Equation S139, gives the CD as:

$$CD = \frac{-\left(\Delta P_{abs}^{i} + \Delta P_{abs}^{c}\right) / P_{inc}}{4T} \qquad \text{(S140)}$$

Equation S140 can be applied to the chiral film in free space as well, with the difference that for individual chiral film $\Delta P_{abs}^{i} = 0$ (no chirality transfer). Thus, the CD of the chiral film can be expressed as:

$$CD_0 = \frac{-\Delta P_{0,\,abs}^{c} / P_{inc}}{4} \qquad \text{(S141)}$$

where we assumed a unity transmittance in the denominator ($T = 1$). $\Delta P_{0,\,abs}^{c}$ is the differential absorbed power of the chiral film in free space, which according to Equation S138 and S139 can be written as:

$$\Delta P_{0,\,abs}^{c} = -P_{inc} \Delta T_0 \qquad \text{(S142)}$$

where the differential transmittance of the bare chiral film is $\Delta T_0 = 4\kappa_0 w \ \mathrm{Im}(\kappa)$,[15] where w is the thickness of the chiral film. Finally, dividing Equation S140 by Equation S142 results in the CD enhancement as:

$$\frac{CD}{CD_0} = CD_{enh.}^{OC} + CD_{enh.}^{CT} \qquad \text{(S143)}$$

where $CD_{enh.}^{OC}$ and $CD_{enh.}^{CT}$ are the enhancements due to optical chirality and due to chirality transfer and defined as:

$$CD_{enh.}^{OC} = \frac{\left(\Delta P_{abs}^{i} / \Delta P_{0,\,abs}^{c}\right)}{T} \qquad \text{(S144)}$$

and,

$$CD_{enh.}^{CT} = \frac{\Delta P_{abs}^{c} / \Delta P_{0,\,abs}^{c}}{T} \qquad \text{(S145)}$$

respectively. The optical chirality term is related to the averaged value of optical chirality over the chiral film by:

$$CD_{enh.}^{OC} = \frac{C_{av} / C_{inc}}{T} \qquad \text{(S146)}$$

References

(1) Orfanidis, S. J. Electromagnetic Waves and Antennas, 2016 [Online]. Available: https://www.ece.rutgers.edu/~orfanidi/ewa/

(2) Bohren, C. F.; Huffman, D. R. *Absorption and Scattering of Light by Small Particles*; John Wiley & Sons, 2008.

(3) Lindell, I. V; Sihvola, A. H. Quasi-Static Analysis of Scattering from a Chiral Sphere. *J. Electromagn. Waves Appl.* **1990**, *4* (12), 1223–1231.

(4) Klimov, V. V; Zabkov, I. V; Pavlov, A. A.; Guzatov, D. V. Eigen Oscillations of a Chiral Sphere and Their Influence on Radiation of Chiral Molecules. *Opt. Express* **2014**, *22* (15), 18564–18578.

(5) Abdulrahman, N. A.; Fan, Z.; Tonooka, T.; Kelly, S. M.; Gadegaard, N.; Hendry, E.; Govorov, A. O.; Kadodwala, M. Induced Chirality through Electromagnetic Coupling between Chiral Molecular Layers and Plasmonic Nanostructures. *Nano Lett.* **2012**, *12* (2), 977–983.

(6) Jackson, J. D. *Classical Electrodynamics*; Wiley, 1999.

(7) Aspnes, D. E.; Studna, A. A. Dielectric Functions and Optical Parameters of Si, Ge, GaP, GaAs, GaSb, InP, InAs, and InSb from 1.5 to 6.0 eV. *Phys. Rev. B* **1983**, *27* (2), 985.

(8) Johnson, P. B.; Christy, R.-W. Optical Constants of the Noble Metals. *Phys. Rev. B* **1972**, *6* (12), 4370.

(9) Mohammadi, E.; Tittl, A.; Tsakmakidis, K. L.; Raziman, T. V; Curto, A. G. Dual Nanoresonators for Ultrasensitive Chiral Detection. *ACS Photonics* **2021,** 8 (6), 1754–1762.

(10) Rahimzadegan, A.; Alaee, R.; Rockstuhl, C.; Boyd, R. W. Minimalist Mie Coefficient Model. *Opt. Express* **2020**, *28* (11), 16511–16525.

(11) Fasman, G. D. *Circular Dichroism and the Conformational Analysis of Biomolecules*; Springer Science & Business Media, 2013.

(12) Migliore, M.; Bonvicini, A.; Tognetti, V.; Guilhaudis, L.; Baaden, M.; Oulyadi, H.; Joubert, L.; Ségalas-Milazzo, I. Characterization of β-Turns by Electronic Circular Dichroism Spectroscopy: A Coupled Molecular Dynamics and Time-Dependent Density Functional Theory Computational Study. *Phys. Chem. Chem. Phys.* **2020**, *22* (3), 1611–1623.

(13) Mohammadi, E.; L. Tsakmakidis, K.; N. Askarpour, A.; Dehkhoda, P.; Tavakoli, A.; Altug, H. Nanophotonic Platforms for Enhanced Chiral Sensing. *ACS Photonics* **2018**, *5* (7), 2669–2675. https://doi.org/10.1021/acsphotonics.8b00270.

(14) Lee, S.; Kang, J.-H.; Yoo, S.; Park, Q.-H. Robust Numerical Evaluation of Circular Dichroism from Chiral Medium/Nanostructure Coupled Systems Using the Finite-Element Method. *Sci. Rep.* **2018**, *8* (1), 1–8.

(15) Mohammadi, E.; Tavakoli, A.; Dehkhoda, P.; Jahani, Y.; Tsakmakidis, K. L.; Tittl, A.; Altug, H. Accessible Superchiral Near-Fields Driven by Tailored Electric and Magnetic Resonances in All-Dielectric Nanostructures. *ACS Photonics* **2019**, *6* (8), 1939–1946.
